# Antimicrobial photodynamic therapy (aPDT) for biofilm treatments. Possible synergy between aPDT and pulsed electric fields

**DOI:** 10.1080/21505594.2021.1960105

**Published:** 2021-09-09

**Authors:** Wanessa de Cassia Martins Antunes de Melo, Raimonda Celiešiūtė-Germanienė, Povilas Šimonis, Arūnas Stirkė

**Affiliations:** Department of Functional Materials and Electronics, Laboratory of Bioelectric, State Research Institute, Department of Functional Materials and Electronics, Center for Physical Sciences and Technology, Vilnius, Lithuania

**Keywords:** Antimicrobial resistance_1_, biofilms_2_, EPS_3_, ROS_4_, aPDT_5_, PEF_6_, photosensitizer_7_ and electroporation_8_

## Abstract

Currently, microbial biofilms have been the cause of a wide variety of infections in the human body, reaching 80% of all bacterial and fungal infections. The biofilms present specific properties that increase the resistance to antimicrobial treatments. Thus, the development of new approaches is urgent, and antimicrobial photodynamic therapy (aPDT) has been shown as a promising candidate. aPDT involves a synergic association of a photosensitizer (PS), molecular oxygen and visible light, producing highly reactive oxygen species (ROS) that cause the oxidation of several cellular components. This therapy attacks many components of the biofilm, including proteins, lipids, and nucleic acids present within the biofilm matrix; causing inhibition even in the cells that are inside the extracellular polymeric substance (EPS). Recent advances in designing new PSs to increase the production of ROS and the combination of aPDT with other therapies, especially pulsed electric fields (PEF), have contributed to enhanced biofilm inhibition. The PEF has proven to have antimicrobial effect once it is known that extensive chemical reactions occur when electric fields are applied. This type of treatment kills microorganisms not only due to membrane rupture but also due to the formation of reactive compounds including free oxygen, hydrogen, hydroxyl and hydroperoxyl radicals. So, this review aims to show the progress of aPDT and PEF against the biofilms, suggesting that the association of both methods can potentiate their effects and overcome biofilm infections.

## Introduction

The over-growing of antimicrobial resistance has been one of the major global health concerns. World Health Organization (WHO) reported that we might return to a time where common infections and minor injuries can kill, causing disastrous consequences over life spans and across generations (1). The hard-won victories against infectious diseases over the last fifty years will be compromised, increasing the hospital stays and public health care costs [2]. Statistics studies from the British Society for Antimicrobial Chemotherapy presume that in 2050 deaths attributable to antimicrobial resistance could be as high as 10 million a year killing more people worldwide than cancer and diabetes combined. This may cost £1 billion to hospital treatment and societal costs in Europe and £66 trillion in lost productivity to the global economy [3].

Antimicrobial resistance is generally associated with the microorganism’s ability to form a biofilm. This microbial community presents several aspects that contribute to biofilm resistance, including reduced metabolic and growth rates, protection by extracellular polymeric substances (EPS), and specific resistance mechanisms conferred by the altered physiology of biofilm compared with planktonic cells [4]. Thus, the current treatments applied to overcome biofilm infections are often inadequate [5]. If the biofilm infection is associated with a medical device, removal of the implant is the best therapeutic alternative (when is possible). However, this strategy can increase patient morbidity and hospital costs [6]. For tissue or sputum-associated biofilms, the only available therapy nowadays is a long-term treatment with high doses of antimicrobials and sometimes a combination of these drugs with different killing mechanisms [6]. Nevertheless, biofilm-growing persists and destroys the infected tissue due to long-term inflammatory response, causing a chronic infection that may lead to sepsis and patient death [7].

As commented above, biofilms show multi-factorial aspects that decrease the efficacy of current antimicrobial treatments. Highlighting the main ones we can include **1) *components of the biofilm matrix***, mainly EPS, that bind with antimicrobials and/or inactive them by enzymes (e.g. beta-lactamases), restricting and hampering the penetration of antimicrobial through biofilms [8]. **2) *Differential physiological activity***, caused mainly by limited oxygen and nutrient penetration through the biofilm. This promotes a low metabolic activity of the microorganisms and consequently decreases the antimicrobial effects, once many of these drugs target processes that occur in microbial growing (e.g. replication and cell wall synthesis) [8]. **3) *Persister cells*** that are microorganisms in a dormant (non-dividing) state, expressing a low metabolic activity that diminishes the susceptibility to all known antimicrobials [9].

The factors described above might lead to the prediction of the “end of the antimicrobial era” [10,11], confirming the urgency to develop new strategies against biofilm resistance. Therapies applying reactive oxygen species (ROS) have been successfully used in chronic and medical device infections caused by biofilm, e.g. antimicrobial photodynamic therapy (aPDT) and pulsed electric field (PEF). ROS presents versatility to be delivered at many clinical sites and a great potential for bio-burden control [12].

aPDT have been shown as a promisor anti-biofilm therapy because the method has multiple targets while presenting a low effect against host cells [13]. Basically, aPDT involves the synergistic combination of a photosensitizer (PS), molecular oxygen and visible light of an appropriate wavelength in order to produce highly reactive oxygen species, which leads to the oxidation of several cellular components and rapid cell inactivation [14]. On the other hand, the PEF is not considered as a powerful approach to produce ROS but can potentiate the aPDT effect in two ways: (I) increasing the PS permeabilization through the cell membrane and biofilm matrix [15], as well as (II) presenting phenomena as electrodissociation of molecules and electrolysis, possibly including ROS formation [16].

This review will be followed by a discussion about the aPDT and PEF effect against biofilms, suggesting the synergism between them to potentate the production of ROS and overcome the main defense mechanisms of biofilm.

## Biofilms

Biofilms have been one of the most significant problems faced for public health, estimating to be responsible for around 80% of all infections, causing many deaths and high health costs worldwide [17]. These problems are directly connected to the biofilm being significantly less susceptible to antimicrobials and host defenses than their planktonic forms [18], tolerating up to 1000 times higher levels of antimicrobials [19].

It has been described that biofilm formation can be found on tissue surfaces (biotic) as well as on medical devices (abiotic) [20]. A variety of indwelling medical devices have been associated with biofilm infection, including urinary/vascular catheters, implants, heart valves and prostheses [21–24]; the intravascular devices followed by urinary catheters and orthopedic implants, are the main causes to nosocomial bloodstream infections [20,25]. Generally, the biofilm infections lead to device malfunction or chemical degradation of biomaterials and consequently require surgical intervention for the implant removal compromising the patient’s quality of life [21].

In addition, biofilm infections commonly manifest as chronic and recurrent diseases [18], and these may not be associated with implanted devices such as chronic airway infections (e.g. pulmonary diseases, wound infections, dental diseases, and so on) and soft tissues infections (e.g. the intestines or lungs) [20].

Regardless of the infection site, a competition between the host cells and pathogenic microorganisms occurs to gain the tissue or biomaterial surface area. Since the pathogen reaches the surface and adheres successfully, they initiate biofilm formation, which alters the microorganism’s virulence properties and protects them against the antimicrobials [26]. These virulence properties, also, allow the microbial cells to survive and grow in adverse conditions, including limited nutrient availability, desiccation, low pH, and so on [27,28].

Thus, biofilm-associated cells present specific mechanisms that lead them to be tolerant or resistant against antimicrobial drugs. Among these mechanisms, we can include the presence of the extracellular matrix (ECM), high cell density that changes the microbial physiological state, and the presence of quorum sensing molecules (QS) and *persister* cells [29,30].

**Figure 1.**
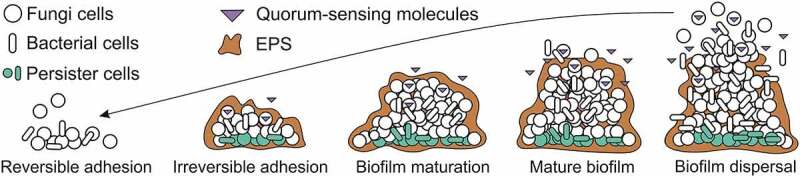
Adapted from [36]: Biofilm formation stages: 1) **Reversible adhesion** occurs when the planktonic cells adhere to a surface area (biotic or non-biotic) through the presence of a few virulence factors (adhesins, pili, flagellum, fimbriae, and glycocalyx) and chemical reactions (van der Waals forces, electrostatic forces, hydrophobic effects), starting the biofilm formation. At this stage, the microbial cells are susceptible to antimicrobials drugs. 2) **Irreversible adhesion**, at this step microorganisms start to grow and replicate, forming colonies that undergo transcriptional modifications for adherence, promoting an exchange of substrate, distribution of important metabolic products, and excretion of metabolic end-products; as well as secrete EPS making the biofilm cells less susceptible to host defense and antimicrobial drugs. 3) **Biofilm maturation**, at this phase the amount of ECM increases around the microcolonies due to continued secretion of EPS and beginning the intracellular communication system through the quorum-sensing molecules (QS), both are important factors of resistance. 4) **Mature biofilm** contains a high concentration of EPS and cavities between it that serve as transport channels of water, nutrients and planktonic cells throughout the biofilm community. 5) **Biofilm dispersal** involves the biofilms detachment due to the restriction of nutrients for the cells. This fact can occur by erosion and sloughing, and the cells search for another surface area to continue surviving.*EPS: extracellular polymeric substrate

This multifactorial complex phenomenon, which is the biofilm, shows five stages of the development cycle: reversible and irreversible attachment, maturation, mature biofilm, and dispersion (Figure 1). During the whole biofilm formation process, the microbial cells express specific phenotype traits that contribute to their virulence and mechanism factors described above [31,32].

The initial contact of the microbial cells to the surfaces (reversible attachment) occurs through the presence of a few virulence factors like cell membrane adhesins, pili, flagellum, fimbriae, and glycocalyx that directly influences the rate of microbial adhesion. In addition, chemical reactions may govern the cells-surface interactions, including van der Waals forces, electrostatic forces, hydrophobic effects, and so on [17,33]. It is important to highlight that microorganisms have more attraction to hydrophobic and nonpolar surfaces, once it reduces the force of repulsion between the cells and the surface [34]. Subsequently, this attachment becomes stable and the microorganisms start to multiply and secrete EPS, forming micro-colonies (irreversible attachment). These colonies undergo transcriptional modifications for adherence, promoting an exchange of substrate, distribution of important metabolic products, and excretion of metabolic end-products [35]. In addition, these new populations will produce a heterogeneous matrix containing 98% of water and a mix of polysaccharides, proteins, nucleic acids, phospholipids, lipids, amyloid fibers, humic substances, and in some cases, surprising amounts of extracellular DNA (e-DNA) [36].

According to Donlan [37], inside the ECM, a channel of water is responsible for bringing nutrients and oxygen to the innermost cells, except for the deep cells named as “dormant cells”. These cells, also called *persister* cells, can be defined as a state in which cells are metabolically inactive and comprise only to <0.1% of the biofilm population [38]. The *persisters* cells are tolerant to the large increases of the antimicrobial concentration having the ability to adapt in adverse environments via “dormancy-growth-proliferation”, and reach this state without undergoing genetic change [39]. This mechanism contributed to a vital role in the microbial biofilm multidrug tolerance, once maintains their survival and cell structure stability [27]. Thus, the *persister* cells can be associated as one of the responsible for the recalcitrance of chronic infections, once it remains viable and regrow the biofilm population after the level of the antimicrobial drops [38,39].

During the biofilm maturation, the concentration of nutrients available determines the final biofilm size [34]. This stage of biofilm formation presents important factors of resistance such as the intracellular communication system that occurs through the quorum-sensing molecules (QS) and the increased secretion of EPS [40].

QS molecules are responsible for a process of cell–cell communication that allow microorganisms to share information about cell density and adjust gene expression [41]. The QS molecules respond to extracellular signaling molecules called auto-inducers (AIs) [42], which accumulate in the environment with the increase of microbial population density [41]. This phenomenon enables the microorganism to track changes in their cell density and gene expression [43,44]. Independently of the microorganism species, the QS systems depend on three basic principles: (I) the production of AIs by microbial community; (II) AIs detection by receptors present in the cytoplasm or cells membrane; and (III) the gene expression activation, as a result of AIs detection and consequently the production of AI [45,46]. Thus, at low cell density, the AIs diffuse away to increase the microbial population density and at high cell density, the cumulative production of AIs leads to molecular and genetic responses, synchronizing the microbial communication [47].

Currently, there are four well-identified and characterized groups of QS molecules: N-acyl-L-homoserine lactone (AHL) QS-system in Gram (-) bacteria, the autoinducing peptide (AIP) QS-system in Gram (+) bacteria, the autoinducer-2 (AI-2) QS-system in both Gram (-) and Gram (+) bacteria and the farnesol, (aromatic alcohol) systems in fungi [48]. These molecules induce signals that control gene expression involved with microbial process including, bioluminescence, sporulation, resistance or tolerance to antimicrobials and evasion to host immune reactions, biofilm formation, and virulence factor secretion (e.g. EPS secretion) [9,49–51]. It is important to emphasize that the inhibition of this QS mechanism may interrupt the biofilm formation, as well as reduce the microbial virulence and resistance [52].

As mentioned above the secretion of EPS is an important virulence factor that confers to the microorganisms both increased antimicrobial resistance and defense from host immune responses [53]. The EPS acts as an adsorbent or reactant reducing the antimicrobials penetration by diffusion limitation and through chemical interactions with the ECM proteins and polysaccharides [54]. In addition, the aggregation of microorganisms into EPS-coated biofilms turns them less susceptible to phagocytosis [35]. The proteins and polysaccharides are the key components of EPS, representing 75–89% of the EPS matrix composition. These components contribute to some functions of EPS matrix, including facilitation of the initial attachment of the cells to different surfaces and protection against several environmental [55].

Biofilm development is a cyclic process because once the cells have established sessile forms they can return to a planktonic form, in case it is more favorable [56]. This process corresponds to the last phase of biofilm formation, named detachment/dispersion (Figure 1). In this stage the disruption of the biofilm occurs, the debris of the biofilm spreads and may cause septicemia. The detachment happens with the decrease of available nutrients promoting biofilm erosion (continual dispersal of a single cell or small portion of the biofilms) or sloughing (large pieces of the biofilms are significantly lost) [57]. According to [34], during the step of dispersion, the biofilm cells produce different saccharolytic enzymes that help to release the surface of the microbes into a new area for colonization.

Important to highlight that the resistance and persistence of the biofilms increase when it is polymicrobial, commonly been associated with chronic infection and occurring in the lung, inner ear, urinary tract, oral cavity, in wounds, and those that are device or foreign body-related [58–60]. Polymicrobial biofilms have a genetic diversity of microorganisms due to an expanded gene pool, which can be more easily shared within the confines of a biofilm community. This may increase the fitness of the residing community, making them more equipped to survive environmental stresses [61]. In addition, several are the advantages that a polymicrobial community (bacterial and/or fungal species) show, including QS system, metabolic cooperation, passive resistance, and many other synergies [58,60]. In this manner, biofilm-community composition and interactions have huge influences on microbial behavior, i.e. the comportment of microorganisms in single species versus multispecies systems is dramatically different [59].

**Figure 2.**
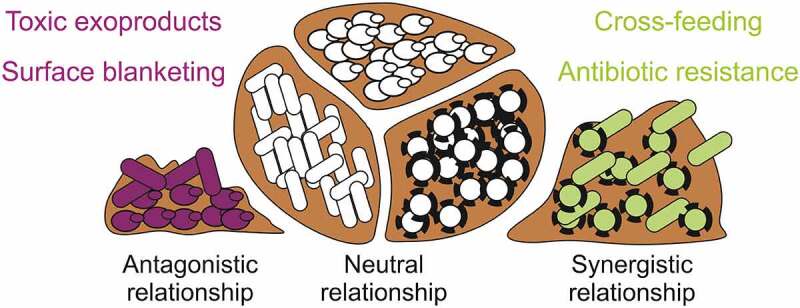
Adapted from [59]: Polymicrobial biofilm interactions. Multiple-species biofilm can be found between the same species of microorganisms (neutral relationship) and between two or more species of microbes such as bacteria and fungi (antagonistic and synergistic relationship). Microbial antagonism produces toxic exoproducts and surface blanketing, while microbial synergy promotes antimicrobial resistance and cross-feeding

Microbial-biofilm interactions are complex and highly dependent on community structure, including the composition and spatial distribution of members within the biofilm population [59] (Figure 2). These interactions can be antagonistic (defined as the suppression of one microbial species by another and/or synergistic (defined as a cooperative interaction between two or more species of microbes that produces an effect not achieved by an individual species alone) [62]. In polymicrobial biofilm infections, the synergism “effects” between microorganisms may include increased microbial growth, enhanced production of EPS, and antimicrobial tolerance, virulence and persistence [63,64]. Metabolic cross-feeding is also a classic cooperative interaction that makes a metabolic “byproduct” enhancing the growth of a neighbor [65]. On the other hand, the antagonistic mechanism is a fierce competition for nutrients and niches between the microorganisms, making them produce virulence factors and chemical signals that can interfere in the behavior or physiology of microbial neighbors [66–68]. This may promote “Surface blanketing” where one species occupies all the attachment sites on a surface, preventing the attachment of another [68,69].

In the literature, have been reported some polymicrobial biofilm interactions between different microorganisms species. *Pseudomonas aeruginosa* and *Candida albicans* are known to form dual-species biofilms that can play extensive roles in nosocomial infections and infection in immunocompromised individuals [70,71]. This occurs cause *P. aeruginosa* attached *to C. albicans* hyphae surface and the Interspecies competition enhances the production of virulence factors and increases mutability, altering the course of host-pathogen interactions infections [70,72,73]. *P. aeruginosa* and *Staphylococcus aureus* coexist in multiple infection settings, being the most prevalent respiratory pathogens in patients with cystic fibrosis and often co-isolated from chronic wounds, including difficult-to-treat diabetic foot ulcers [74–76]. Their interactions mechanisms still are poorly understood, but some studies showed that secreted products of *P. aeruginosa* could enhance biofilm tolerance of *S. aureus* to vancomycin by 100-fold, through the function of the electron transport chain and slowing growth of this gram-positive bacteria [77].

[69] reproduced mixed-species biofilm comprising *P. aeruginosa, Pseudomonas protegens* and *Klebsiella pneumoniae* to study how interspecies interactions affect biofilm development, structure and stress responses. The authors reported that the mixed-species biofilm exhibited distinct structures, presenting a delay of 1–2 days compared with the single-species biofilms. The composition and spatial organization of polymicrobial biofilm changed along the flow cell channel, where nutrient conditions and growth rate of each species could have a part in community assembly. In addition, the microorganisms in a polymicrobial biofilm showed to be more resistant to antimicrobials sodium dodecyl sulfate and tobramycin than the single-species biofilms. Suggesting, community-level interactions are unique to the structured biofilm community, where the members are closely associated with each other.

Thus, biofilms mechanisms of resistance, multiple or single species ones, limit the effectiveness of conventional antimicrobial drugs, making urgent the development of new strategies a target of scientific attention [34].

Nowadays, several anti-biofilm approaches have been proposed, focusing on the inhibition of biofilm adhesion and growth (Table 1). The alterations of the medical device materials are one of the most studied by researchers in order to prevent biofilm formation. Several are the physical-chemistry techniques [36] (e.g. ion-beam, plasma-technique, surface photo-grafting) and the materials used [78] (e.g. polyvinylchloride, polyester, chitosan, polystyrene) to produce these new devices. In addition, electrical approaches [79], ultrasound [80], nanoparticles [81], liposomes [82], and polymer-based drug delivery vehicles [83] have been applied against the biofilm, aiming to increase the penetration through the surface of this microbial community and toward deeper cells.

**Table 1.** Effect of different approaches against microbial biofilms

Approaches	Microorganisms	CFU (log_10_) reduction	References
Nanoparticles	*Staphylococcus aureus*	4.13	(84)
	*Escherichia coli*	5.32	(84)
Photo-grafting	*Candida albicans*	6.44	(85)
	*E. coli*	5.00	(86)
Furanones	*Pseudomonas aeruginosa*	2.55	(87)
RNA III	*S. aureus* MRSA	4.58	(88)
	*S. aureus* MSSA	4.64	
	*S. aureus*	4.00	(89)
Ultrasound	*S. aureus*	4.00	(90)
	*E. coli*	4.85	(91)
DNAse	*P. aeruginosa*	3.20	(92)
	*Enterococcus faecalis*	6.40	
	*Salmonella typhimurium*	5.60	
	*S. aureus*	7.20	
Plant extraction	*S. aureus*	5.00	(93)
	*Klebsiella pneumoniae*	3.08	(94Sánchez et al., 2016)
	*E. faecalis*	2.7	(Sánchez et al., 2016)
	*E. coli*	3.00	(Sánchez et al., 2016)

* Table shows few studies that have been realized to find an alternative approach to overcome the microbial biofilm.

**Figure 3.**
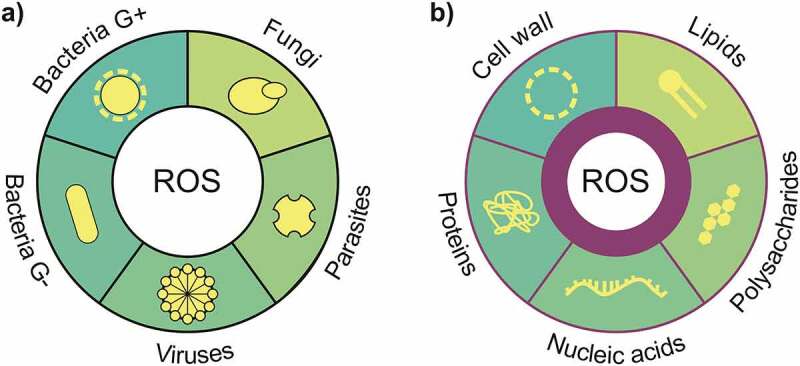
Adapted from [97]: ROS presents (a) a wide therapeutic window that affects different species of microorganisms (bacteria, fungi, virus and parasites) and (b) it is a nonselective multiple target oxidizing various biomolecules, promoting substantial cell damage

Despite the large number of therapies being practiced to achieve biofilm killing, generally, they focus on a specific microorganism or aims at one specific target of the biofilm structure [95]. ROS can be a novel solution to overcome these factors once shows a wide therapeutic window that affects different species of microorganisms (bacteria, fungi, virus and parasites), are nonselective with multiple targets in focus and known to oxidize various biomolecules, promoting substantial cell damage [96,97] (Figure 3). Therefore, therapies that produce ROS can be a great potential to reduce biofilm antimicrobial resistance.

## Bio-effects of aPDT and PEF

### Antimicrobial photodynamic therapy

aPDT is a minimally invasive method that has been reported as an alternative treatment of infections caused by several pathogens [36]. This therapy presents some advantages that can overcome antimicrobial resistance problems, including a broad spectrum of action, the efficient inactivation of multi-antibiotic-resistant strains, the low mutagenic potential, and the lack of selection of photo-resistant microbial cells (98).

aPDT has been further studied, for the past several decades, showing to be effective against biofilm of Gram-negative and Gram-positive bacteria and fungi, that can be multiple or single-species biofilm [99]. Several authors have been reported that aPDT-generated ROS has many possible targets on biofilms, mainly against EPS matrix [99] that includes the DNA [100], lipids [101], proteins [102,103] and extracellular polysaccharides [104]. In addition [105], showed the efficacy of the aPDT against several stages of biofilm development. Thus, they designed experiments to analyze the effect of different light doses (4.23; 8.46; 12.70; 16.93 and 21.16 J/cm^2^) against several ages of *S. aureus* biofilms (0; 6; 11; 17; 24; 32; 40 and 48 hours). As result, optical density analysis showed the most optimum biofilm reduction happened when biofilm age is perfectly constructed (about 17 hours) and with 91% reduction; as well as the longer biofilm age lived among those biofilms, the greater the reduction.

[106] studied the aPDT effect combined with usual endodontic therapy, against the Gram‐negative bacteria, *Proteus mirabilis* and *P. aeruginosa* formed at root canals. Bioluminescence imaging was used to periodically quantify bacterial burdens and the treatment effect. The aPDT study was performed using a conjugate between polyethylenimine and the PS chlorin (e6), as a light source was used a 660‐nm diode laser. They realized that this combination reduced the bioluminescence by >98% and the bacterial regrowth after 24 hours of the treatment [107], also combined the endodontic therapy with aPDT using Zn(II)chlorin e6 methyl ester (Zn(II)e_6_Me) activated by red light against monospecies against mixed biofilms of *Enterococcus faecalis* and *Candida albicans*. The results showed that Zn(II)e_6_Me once activated was able to remove around 60% of the biofilm biomass.

The chlorin (e6) have been used as PS for several aPDT application, as pediatric otitis media caused by *Moraxella catarrhalis, Streptococcus pneumoniae*, and nontypeable *Haemophilus influenza* [108]. demonstrated that the chlorin (e6) elicits significant bactericidal activity against both planktonic cultures and established biofilms formed by these three major pathogens (with an efficacy of ≥99.9% loss of viability) [109]. performed a study applying Photodithazine® (commercially available cationic chlorin-*e*6) against multispecies biofilms from *Streptococcus mutans, C. albicans* and *Candida galbrata*. After being exposed to red light from a LED light source aPDT-treatment showed reductions of 1.0 or 2.0 log_10_ for *Candida* spp. or *S. mutans*, respectively. Concerning porphyrins, other tetrapyrrolic PS, upon irradiation have been reported to inactivate about 4.0 log_10_ of the *P. aeruginosa* biofilm [110], reduced 5.0 log_10_ of *C. albicans* biofilm [111] and up to 2.0 log_10_ of *Sthaphylococcus aureus* biofilm [112].

[113] evaluated the efficacy of aPDT mediated by chloro-aluminum phthalocyanine encapsulated in cationic nanoemulsions (ClAlP-NE) to treat oral candidiasis *in vivo* and its effect to prevent the *C. albicans* biofilm formation. aPDT was as effective as Nystatin reducing 1.4 and 2.0 log_10_ of the cell viability, respectively, meaning that both treatments reduced the ability of *C. albicans* to adhere on a surface and form biofilm. Later [114], showed that the oxidative stress caused by aPDT affects the expression of *C. albicans* genes related to adhesion and biofilm formation (ALS1 and HPW1) and oxidative stress response (CAP1, CAT1, and SOD1). In addition, (115) reported the potential of chlorophyll extract of papaya leaf (0.5 mg/L) as an exogenous photosensitizer against *C. albicans* biofilm. Thus, upon irradiation by diode laser at 445 nm and 650 nm the *C. albicans* biofilm was reduced to 25% and 32%, respectively.

The phenothiazinium has been, extensively, applied in aPDT studies, mainly the dyes methylene blue and toluidine blue (TBO). Both are designated to be potential efflux pump substrates in a variety of microbial species [116,117], enhancing the effect of the photodynamic inactivation on Gram positive species [118,119]. showed the aPDT effect against polymicrobial biofilms of *P. aeruginosa* and methicillin‐resistant *S. aureus* (MRSA). They mimic, *in vitro*, chronic recurrent sinusitis and applied the methylene blue as photosensitizer and 670 nm non–thermal‐activating light, which results in 99.99% of biofilm reduction [120]. showed the aPDT action against antibiotic-resistant polymicrobial biofilms of *P. aeruginosa* and MRSA grew in endotracheal tube. With a methylene blue photosensitizer and 664 nm non‐thermal activating light they obtained biofilm reduction around >99.9% (*P* < 0.05%) after a single treatment.

It was evaluated by [121] the synergisms effect of laser diodes 830 nm (as the light source) and Toluidine blue O (TBO) on the following periopathogenic bacteria: *Aggregatibacter actinomycetemcomitans, Porphyromonas gingivalis, Fusobacterium nucleatum* and *Prevotella intermedia*. After the irradiation, the microorganism suffered aPDT effect, mainly the *A. actinomycetemcomitans* and *P. intermedia* that were 100% reduced. The photo-effect of toluidine blue was also evaluated against several strains of MRSA and methicillin-sensitive *S. aureus* (MSSA), isolated from burns patients. Upon irradiation by red LED (630 ± 10 nm) the TBO significantly reduced the mean cell survival in the MRSA (2.5–3 log_10_) and MSSA (2.75–3.1 log_10_) isolates [122,123]. verified the effect of two phenothiazinium dyes (TBO and new methylene blue (NMB)) against *S. mutans* biofilm. TBO was found to have a better antibacterial as well as an anti-biofilm effect than NMB. They correlated this result with the highest production of singlet oxygen production by TBO than NMB, even the NBO can produce free radical (HO^∙^).

Another PS that showed aPDT effect against *S. mutans* biofilm was the curcumin that even in low concentrations (above 3 g/L), after irradiation by LED (central wavelength: 450 nm; light dose: 5.7 J/cm^2^) caused a 3 log_10_ of bacteria reduction [124]. In addition [125], compared the effect of rose bengal and erythrosine against *S. mutans* and *Streptococcus sanguinis* biofilms. These PSs at a concentration of 5 μM and after irradiation of blue LED (455 ± 20 nm) for 180 s, caused a reduction of 0.62 and 0.52 log_10_ CFU mL^−1^ for *S. mutans* biofilms (*p* = 0.001), and 0.95 and 0.88 log_10_ CFU mL^−1^ for *S. sanguinis* biofilms (*p* = 0.001), respectively.

An interesting PS is the perinaphthenones (phenalenones) which is a BODIPY dye used as a reference standard for the generation of ^1^O_2_ [126,127]. modified this PS to SAPYR [2-((4-pyridinyl)methyl)-1 H-phenalen-1-one chloride], introducing a batch of positively charged derivatives based on a 7-perinaphthenone-structure to increase the aPDT effect. They verified the SAPYR anti-biofilm properties against *E. faecalis* and *Actinomyces naeslundii* after the irradiation of blue LED. The authors concluded that this PS has two mechanisms of action against biofilms, including the disruption of biofilm structure without illumination; and after irradiation, it inhibits the polymicrobial biofilm after one single treatment with efficacy of ≥99.99%. Later, (128) showed that the effect of SAPYR against the same bacteria-biofilms (*E. faecalis* and *Actinomyces naeslundii*) can be increased by the application of a formula specific to adjust the number of photons absorbed by PS after the irradiation. In addition, the authors exhibited that aPDT effect of MB is smaller or none compared to SAPYR. Recently, it was compared the aPDT effect of two phenalen-1-one derivatives (SAPYR and SAGUA) against a polymicrobial biofilm commonly found in periodontal disease. As result, SAPYR reduced up to 6.1 log_10_ of CFUs while SAGUA was less effective inhibiting up to 2.8 log_10_. In addition, the flow cytometric analysis revealed no damage of cytoplasmic membranes after aPDT with both phenalen-1-one derivatives. Besides that this experiment is the first step to get understandings of the mechanism and damage patterns of this PS class in aPDT [96].

As commented above, polymicrobial biofilms can be inhibited by aPDT [129]. applied the following conditions, MB (500 μg/ml), red light (664 nm) of 150 mW/cm^2^ with a light dose of 216 J/cm^2^, against the biofilm of multidrug-resistant *P. aeruginosa* (clinical isolate) and MRSA clinical isolate that was 99% reduced [130]., reported the effect of Photodithazine (125 mg/L) and red light (660 nm, 25 mW/cm^2^, 37.5 J/cm^2^) against the multiple-species biofilm of *C. albicans, C. tropicalis* and *C. glabrata*, inhibiting to 0.9, 1.4 and 1.5 log_10_, respectively [104]. incubated the polymicrobial biofilm of *S. aureus* and *C. albicans* with the PS Tetra-Py^+^-Me (20 µM) and after irradiation by white light with (64.8 J/cm^2^), inhibited 6.5 log_10_ and 4.6 log_10_, respectively. Duo-species biofilm of MRSA and methicillin-resistant *S epidermidis* was inhibited up to 80% to 90% by PS ALA (40 mM) after irradiation by red light (635 nm) at 300 J/cm^−2^ [131].

#### Fundamental mechanisms

Basically, aPDT involves the synergistic combination of a photosensitizer (PS), molecular oxygen and visible light of an appropriate wavelength in order to produce highly reactive oxygen species (ROS), which leads to the oxidation of several cellular components and rapid cell inactivation [36].

ROS are radical or molecular species of oxygen that are in a more reactive state than molecular oxygen and can be reduced. Molecular oxygen contains two unpaired electrons with parallel spin configurations in its outer shell. Because of this spin restriction, one-electron redox reaction takes place with other atoms or molecules, which results in several high-reactive intermediates, such as superoxide anion (O_2_^∙−^), hydrogen peroxide (H_2_O_2_), and hydroxyl radicals (HO^∙^):

(1) $$O_{2}\overset{e^{-}}{\to }O_{2}^{-}\overset{e^{-}+2H^{+}}{\to }H_{2}O_{2}\overset{e^{-}+H^{+}}{\to }HO^{\;}\overset{e^{-}+H^{+}}{\to }H_{2}O$$

Due to their high reactivity, free radicals can abstract electrons from other compounds to attain stability. The attacked molecule thus loses its electron and becomes a free radical itself, beginning a chain reaction cascade, which could finally affect the living cell. Cellular ROS are generated endogenously mainly during the process of mitochondrial oxidative phosphorylation, in which molecular oxygen is reduced to water in the electron transport chain. The superoxide radical O_2_^•−^ is produced at several sites in the mitochondria, then it is converted to H_2_O_2_ or HO^∙^ by various enzymes. The production of various ROS also appears in other organoids, such as peroxisomes or endoplasmic reticulum, also because of the normal metabolism of various cellular enzymes, such as NADPH oxidase and others. ROS may arise from exogenous sources, such as xenobiotic compounds, ultraviolet light, ionizing radiation, pollutants, electrical pulses as well.

**Figure 4.**
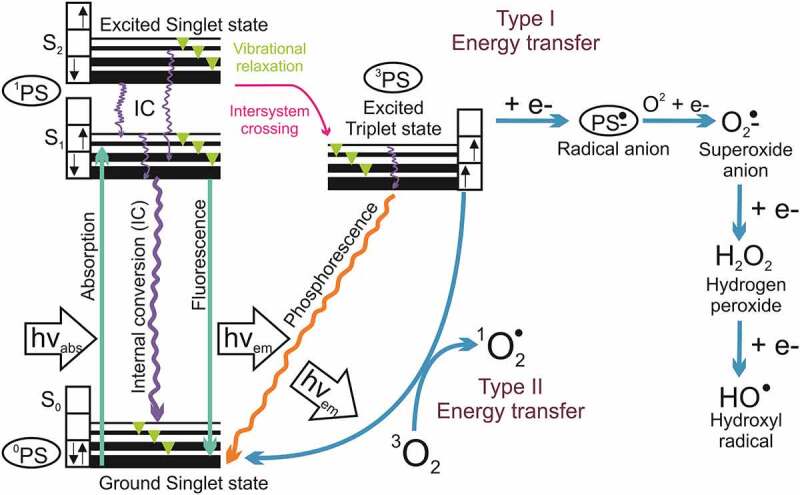
Adapted from [10]: Jablonski diagram showing the photochemical and photophysical mechanism of aPDT

ROS are thought to play a dual role in cells. They are required for maintenance of physiological cell functions, including proliferation, of the stem cells via stimulation of specific target proteins, signal transduction, hydrogen peroxide is the most important signaling molecule of redox metabolism, host defense (phagocytosis), but are also often associated with the oxidative stress [132,133]. Human tissues have a substantial ability to tolerate ROS under normal conditions. Nevertheless, when the production of ROS exceeds the capacity of antioxidant defenses, oxidative stress is inflicted, which leads to harmful effects on the function and structural integrity of lipids, proteins, and DNA [132–134].

(Figure 4) shows Jablonski diagram portraying the aPDT mechanism: after irradiation, the PS transfers from its ground state singlet (lowest energy level, ^1^PS) to a short-lived excited singlet state (^1^PS*). The ^1^PS* fastly loses energy by the following process: (I) radiative by fluorescence or (II) non-radiative decay pathways by internal conversion-heat. The vibrational relaxation (VR) is also an important process involved in aPDT mechanism, in which an electron in a high vibrational level of an excited state can fall to the energetically lowest level of that state (S1) and the energy will be dissipated as heat [135]. If an electron has been boosted to a higher energetic stated, after VR, it will fall for the first excited singlet state. Fluorescence emission promotes molecular relaxation to S0 and always starts from the lowest level of S1 [136].

*PS: photosensitizer; O_2_: molecule oxygen; ^3^O_2_: ground state oxygen; superoxide anion: O_2_^•−^; hydrogen peroxide: H_2_O_2_; hydroxyl radicals HO^•^ and ^1^O_2_ singlet oxygen

On the other hand, a fraction of ^1^PS* molecules can be converted to a longer-living excited triplet state PS (^3^PS*), where two electrons are unpaired and have the same spin, through a non-radiative process called intersystem crossing (ISC). This process promotes a “spin-forbidden transition” that violates the rule of no spin change during a change of an electronic state. As a result of spin-orbit coupling, such transitions take place with a certain probability. In addition, occurs the phosphorescence process (emission of an electromagnetic quantum) after a rapid VR within ^3^PS* energetic level and a radiative relaxation to S0 [135,137]. In other words, ^3^PS* can decay back to the ground state by emitting a phosphorescent photon or by mechanisms generating ROS (Type I and II) [10,96,138].

The ^3^PS* has a much longer lifetime (µsec) than ^1^PS* (nsec), providing it a satisfactory time to interact, specifically, with a molecule in its electronic triplet ground (as dioxygen,^3^O_2_) once interactions between triplets and singlets are spin-forbidden [10]. This long lifetime of 3PS* enables it to transfer its energy by colliding to molecular oxygen (O_2_), leading to the produce of ^1^PS and oxygen singlet (^1^O_2_) molecules, this process is named Type II photochemical process [138]. In addition, the 3PS* can undergo a chemical process, named as Type I pathway, occurring electron transfer reactions that form ROS (superoxide O_2_^•−^, hydrogen peroxide H_2_O_2_ and hydroxyl radicals HO^∙^) [14]. The superoxide is produced through a reaction between a radical anion (PS^−*^) and ground state oxygen. The O_2_^•−^ undergoes a dismutation or one electron reduction forming H_2_O_2_, which is the precursor of HO^∙^ formed via Fenton-like reaction (Figure 4) [96].

The PS returning to its ground state (^1^PS) may permit that a new photochemical cycle happens since it is ready to absorb a new photon and generate more ROS. According to [96], one molecule of PS has enough lifetime to produce thousands of oxygen singlet molecules. The ^1^O_2_ is the most reactive ROS and, consequently, it has a short lifetime of 3–4 ms that promotes a no longer diffusion of 0.3 mm. Its short life can be associated with a physical and chemical quenching that occurs by collision or reactions with surrounding molecules, respectively [10,96,139,140]. The ^1^O_2_ lifetimes depend on the surrounding medium, and it has been described that is much longer in lipophilic environments than an aqueous medium [10].

**Figure 5.**
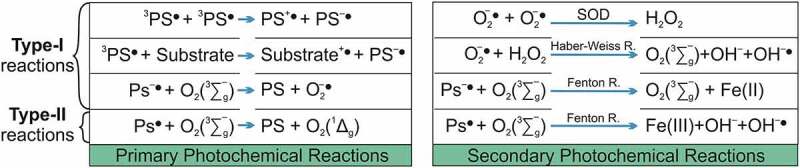
Adapted from [135]: Overview of photochemical reactions during aPDT represented by primary and secondary photochemistry reactions that produce ROS, promoting cytotoxic reaction that causes cellular damage

Summarizing (Figure 5), aPDT photochemistry reactions to promote cellular damage (cytotoxic reactions) are defined as primary (type I and II mechanisms) and secondary (SOD, Haber Weiss and Fenton reaction) [135]. Type-I photochemical reaction may form radical anion or cation by transfer of electrons (or protons) to molecular oxygen or other adjacent molecules, respectively [137]. Superoxide anions are formed by the transfer of an electron from the PS to molecular oxygen [141]. Despite these anions being not very reactive in biological systems, they easily react with H_2_O_2_ that in turn is quite relevant in promoting cellular damage, mainly because is not restricted to one cellular compartment [142].

* H_2_O_2_: hydrogen peroxide; O_2_ (^1^Δ_g_): singlet oxygen (excited state); O_2_^3^ ∑^−^_g_: triplet oxygen (ground state); O_2_^−•^: superoxide anion; OH^−•^: hydroxyl radical; SOD: superoxide dismutase; X^−/+^: anion/cation species; X^•^: radical species.

During the energy transfer to molecular oxygen from a type-II photochemical reaction, the very reactive ^1^O_2_ is formed [135,141]. This oxygen species is an uncharged molecule that diffuses through the cytoplasm and biological membranes [142]. The lifetime of ^1^O_2_ modifies according to the localization and medium, in the cytoplasm due to the presence of reacting molecules it is reduced by more than one order of magnitude, max 1 × 10^−7^sec [143]. On the other hand, the diffusion constant for singlet oxygen in cells is 1.4 × 10^−5^ cm^2^.s^−1^ [144].

Both type-I and type II photochemical reactions occur in parallel, and at higher concentrations of hydrogen peroxide, occurs Haber Weiss reaction, where it reacts with superoxide anions to form the very reactive hydroxyl radical. In addition, hydrogen peroxide in a medium with a redox potential of E_0_ equals 1.35 V shows very low activation energy and can attack and oxidize any molecule within a cell [135].

Thus, ROS’s short lifetime diffusion distance hinder reaches pathogens localized far from where the ROS are produced [10,145]. For most photosensitizers employed in aPDT, the type-II photochemical reaction represents the dominant process [146]. As a consequence, the intracellular localization of the PS greatly determines the site of cellular damage set by aPDT (Figure 3 shows some of the ROS targets). These factors highlight the importance of PS diffusion through the EPS to reach the deeper layer of the biofilm and bind to the microbial cells, mainly the *persister* cells. Another important challenge of aPDT is that bacteria may upregulate the expression of some enzymes, contributing to their adaption toward ROS generated from the type I mechanism. On the other hand, if the distance between the oxidative burst and these defense enzymes is too long, it will not help the bacteria to overcome the aPDT [147,148].

Recently, a Type III photochemical pathway has been proposed, which implies an oxygen-independent photoinactivation of the microorganism, although the “photodynamic” term by definition involves oxygen [10]. This process involves photoinduced electron transfer that produces reactive inorganic radicals. The authors assume that this mechanism may occur in anaerobic or hypoxic tissue infections, where the O_2_ concentration is below 0.5% [10]. They justify this hypothesis once some studies have been shown the increase of the aPDT efficacy in the absence of oxygen. The PSs psolarens and tetracyclines are examples of that and the addition of organic salts, mainly potassium iodide and sodium azide, have potentiated the bacterial killing of aPDT [10].

#### Photosensitizers

The aPDT effect depends on PS localization and accumulation in diseased tissue as well as the amount and time of ROS produced after the light delivery [138]. The efficiency of aPDT strongly depends on PS concentration and its Physico-chemical properties as well as the irradiation time (depends on the light source) and the microorganism morphology [149,150].

In addition, an ideal PS would be a chemically pure drug with specific uptake by the target tissue, should have a substantial triplet quantum yield to promote a high production of ROS, and do not present dark toxicity (i.e., activated only upon irradiation) [151,152]. Another important point is the rapid clearance of the PS from non-infected tissues, minimizing the unique side effect of the aPDT, phototoxicity [138,153]. The chemical stability on shelves and in solvents commonly used in pharmaceutical preparations (e.g. saline solution) is an important factor for PS activity; thus, amphiphilic PS could show the requirements for aqueous dissolution and hydrophobic moieties to enable it to traverse cell membranes [151]. The low cost and easy production of the PS on large scales are also very important.

The “optical window” for biological tissue is a relevant aspect of the PS properties, meaning high photonic absorption of the PS in the far red/near-infrared portion of the electromagnetic spectrum. This range (550–700 nm) is ideal once water absorbs infrared energy (>750 nm) and ultraviolet light (100–400 nm) is deleterious to cell homeostasis, melanin, heme, and other endogenous biological pigments absorb shorter wavelengths [151].

Although it is already known the properties of an ideal PS, to date no one presents all. On the other hand, several of them are available to be applied in aPDT and they are classified by generations. The first generation of PSs includes Photofrin, which is a mixture of porphyrin oligomers. The second generation of PSs includes improved and purified synthetic tetrapyrrole derivatives, including meso-tetra-hydroxyphenyl-chlorin, 5-aminolevulinic acid. The third generation, which is a combination of second-generation PSs and a drug delivery carrier, e.g., antibodies or liposomes [154].

According to several authors, small PS with cationic charges and an asymmetric structure can reach a broad spectrum of microbial inactivation [10,155]. In general, Gram-positive bacteria and yeasts are easily affected by neutral and anionic PS, while Gram bacteria were not [99]. Gram-negative bacteria have a complex structure that is many-layered containing strongly negatively charged lipopolysaccharides (LPS), lipo-proteins, and proteins with porin function, making this pathogen more susceptible to cationic PS [13]. This fact has been explained due to cationic PS “self-promoted uptake pathway”, where it shows higher affinity to anionic binding sites associated with surface LPS molecules than the normally bound divalent Ca^2+^ and Mg^2+^ cations [156, 157, 158]. Thus, the metal ions reorganize the bacteria membrane allowing the diffusion of the PS and consequently increasing the concentration of PS inner the cytoplasm [36,156,157].

Gram-positive species are easily killed by any type of PS, once has a porous cell wall (peptidoglycan and lipoteichoic acid) allowing the PS to reach the cytoplasmic membrane [159,160].

Despite the fungi confer more specialized organelles than Gram-negative bacteria and a rigid cell wall, have been evidenced that they are more susceptible to aPDT [161]. Activated PS induces alterations on cytoplasmic membrane of fungi, causing damage to their organelles as lysosomes, mitochondria and nucleus that lead to fungal death [162,163].

Fungi and bacteria organized as a biofilm, normally, hinder the PS permeabilization through EPS to reach microbial cells, decreasing the aPDT effect. Some studies, however, showed that the PS can be totally sequestrated by EPS and bind to biofilm matrix [164,165]. Between the EPS components, the polysaccharide, proteins and DNA are the most abundant, attracting more attention to be photodamaged. A study showed that only the PS Tetra-Py^+^-Me promoted an EPS reduction of around 30%, once its interaction with the matrix caused a polysaccharide detachment. Upon irradiation the polysaccharide level was further reduced up to 80%, reducing 2.8 log_10_ of the microbial cells [104].

Damage caused to protein and DNA by aPDT significantly reduces the biofilm cellular metabolism and may disrupt the biofilm structure [103]. Hematoporphyrin has been known to break single-stranded and double-stranded DNA and to promote the disappearance of the super-coiled fraction of plasmid DNA in bacteria species [166]. Other powerful PS as Pc 4, promoted microbial nuclear damages, leading the *C. albicans* to exhibit characteristics of apoptosis [167]. While some less powerful PS caused non-lethal damage to DNA that may be repaired by DNA repair systems [168].

On the other hand, these less powerful PSs can easily photodamage the proteins once they are an abundant target and shows a rapid reaction rate with ROS. The sulfur-containing amino acids, cysteine, and methionine are more susceptible to ROS. The ROS reaction on amino acid residues may cause the production of dityrosine moieties, form the protein-centered and ROO^∙^ radical, as well as cleave peptide bonds. Thus, affects the metabolic activities of the proteins, interfering directly in the biofilm and cells development [99,102,169].

In addition, some authors affirmed that cationic PS presents more effectiveness do bind and inhibit the biofilm than anionic PS. This may be explained by the EPS “trapping”, i.e. outside of the cell occurs ionic and hydrophobic interaction with the anionic PS decreasing the PS able to site the plasma membrane, considerate as one of the most important aPDT-target [36,170].

The chemical structure of the PS also has an influence on the photochemical mechanisms pathways and the ratio of ROS produced [171]. (Table 2). According to [172], Gram-positive bacteria and fungi are more susceptible to ^1^O_2_, while Gram-negative bacteria are more sensitive to HO^∙^. Suggesting that Type II mechanism have more effect against Gram-positive bacteria and fungi while Type I shows more influence on Gram-negative. Important to highlight that both mechanisms happen during the aPDT, but depending on PS and microorganism involved one of the mechanisms may be more effective than another one.

**Table 2.** Photosensitizers and reactive oxygen species (ROS) produced by them

Photosensitizer	Type of ROS produced, predominantly	References
Phthalocyanines	^1^O_2_	[138]
Porphyrins	O_2_**^·-^**, H_2_O_2_, and **^·^**OH	(173)
Chlorins	O_2_**^·-^**, H_2_O_2_, and **^·^**OH	[96]
Bacteriochlorins	O_2_**^·-^**, H_2_O_2_, and **^·^**OH	[138]
Phenothiazinium (MB, TB and NMB)	^1^O_2_	[174]
Rose Bengal	^1^O_2_	(175)
Eosin and Erythrosine	^1^O_2_	(176)
Hypericin	^1^O_2_ and O_2_**^·-^**	(177)
Curcumin and it derivatives	O_2_**^·-^**, H_2_O_2_, and **^·^**OH	[192,193]
Riboflavin and it derivatives	O_2_**^·-^**, H_2_O_2_, and **^·^**OH	[191,194]
Fullerenes	Hydrophobic environments: ^1^O_2_ Hydrophilic environments: O_2_**^·-^**, H_2_O_2_, and **^·^**OH	[205]
Phenalenones	^1^O_2_	[127]

* Table shows the main ROS (superoxide anion: O_2_^•−^; hydrogen peroxide: H_2_O_2_; hydroxyl radicals HO^∙^ and ^1^O_2_ singlet oxygen) produced by each PS, however is important to emphasize that the PS are capable to produce all type of ROS. MB: methylene blue; TB: toluidine blue; NMB: new methylene blue.

In biofilm cases, HO^∙^ and ^1^O_2_ are the two most commonly found to attack diverse components of biofilm matrix, cell surface and inside the cells. Large amounts of ROS may oppress the antioxidant defenses of microbial cells, resulting in the collapse of the biofilm matrix and disintegration of microbial cells [165]

Given the above, oxidative targets of aPDT mostly depend on the respective localization of the PS and its chemical structure as well as on the ROS diffusion lifetime. For a better understand, in the following, the main classes of PS are described shortly (Table 2).

The PS can be classified accordingly to some properties and characteristics that they similarly present, for example, be synthetic or natural dye. Among the synthetic PS, phenothiazinium salts and the rose Bengal have shown better results against microbial infections. Phenothiazinium as methylene blue (MB) and toluidine blue (TB) is effective PS that presents at least a single positive charge and present a low ^1^O_2_ quantum yield (0.4), acting mainly as stated by Type I process [96,174]. In addition, they present an excellent light absorption property (600–690 nm) to treat local microbial disease and exhibit low toxicity levels in mammalian cells [174,178]. Besides MB and TB, new phenothiazinium structures have been improved and applied against dental caries, in oral candidiasis, stomach and leg ulcers [179,180].

Rose Bengal is an anionic water-soluble xanthene dye that absorbs in a visible light range of 480–800 nm [181]. This PS exhibits a good quantum yield of singlet oxygen production (0.6 to 0.8) [182] and increases its triplet yield with the addition of heavy atoms into the ring structure [138]. Studies have shown the effect of RB in aPDT against biofilm of different bacteria species, such as *S. aureus, Listeria innocua, Enterococcus hirae* and *E. coli* [183], and fungi as *C. albicans* [184]. Eosin Y and erythrosine are also xanthene dyes that show intensive absorption between 480 and 800 nm (green spectrum) and act mainly according to type II mechanism; however, their negative charges decrease the aPDT efficiency [96,126].

Natural products have been extensively applied in several medical therapies and this fact is not different for aPDT. Hypericin, riboflavin and curcumin are natural PS, commonly, used in aPDT to disrupt microbial biofilm [185–187]. Hypericin, a polycyclic quinone extracted from *Hyperforin perforatum* (St. John’s wort) that has a maximum absorption peak of ~599 nm [188]. It shows a substantial quantum yield, intense absorption spectrum in the visible region, low photo bleaching, short half-life and a wide excitation range; all significant advantages for aPDT applications [189,190]. Riboflavin also called vitamin B12, is a very poor hydrophilic PS that shows two absorption peaks 360 nm (UVA) and 440–470 nm (blue spectrum) [96]. This PS derivatives with positive charges exhibit a considerable improvement of aPDT effect, mainly due to the increase of oxygen singlet quantum yields up to 0.8 [191]. Curcumin is a hydrophobic polyphenol found in the rhizome of turmeric (*Curcuma longa* L.) that presents an absorption spectrum between 300 and 500 nm with a relatively high extinction coefficient, contributing to increase the ^1^O_2_ generation [192]. Hydrogen peroxide and hydroxyl radical are the others main ROS that curcumin-derived photolytically produces [192], acting accordingly to Type I mechanism [96]. Important to highlight that have been reported chemical modification in curcumin structure, as the addition of positive charges, to overcome its hydrophobicity [193].

Bacteriochlorins can present as natural or synthetic tetrapyrrolic derivatives, which have two reduced pyrrole rings and these reduced centers are diagonally opposite [195]. This PS can be effective at lower concentrations, once has high molar absorption coefficients in the therapeutic window (600–800 nm) [196]. Moreover, have been shown photostability, long-lived triplet states, and high quantum yields in the generation of ROS [197].

Porphyrins, chlorins, and phthalocyanines are also heterocyclic-macrocyclic compounds that consist of four pyrrole cycles (porphyrins, phthalocyanines) or three pyrrole and one pyrroline subunits (chlorins) [96]. Chlorins and porphyrins are most efficiently activated at Soret band (~405 nm) and present small peaks at Q bands (500 nm) [138]. These PSs exhibit single oxygen quantum yields between 0.5 and 0.8 structure, acting predominantly according to Type II mechanism [96]. In addition to their eight positive charges, has been reported its chemical modification adding more cations to increase the aPDT efficacy [110,198].

On the other hand, phthalocyanines are hydrophobic and uncharged photosensitizers that have generally been applied to photodynamic field, being necessary to attach different substituents at its peripheral position, as cationic charges [199]. These positive charges affect amphiphilic character of this PS, increasing the effect against microbes [126]. The phthalocyanine shows strong absorption in far red-light in the wavelength 600–800 nm and photo-physical properties such as quantum yield of singlet oxygen that can be enhanced by conjugating the phthalocyanine with metal nanoparticles such as Ag, Zn, Co and Fe [200,201]. Zinc phthalocyanine has been extensively applied against biofilm due to its high molar extinction coefficient and singlet oxygen quantum yield of 0.67, as well as exhibit photochemical properties more stable than other phthalocyanines [202].

Nanoparticles also have been used as an alternative not only to increase the solubility of some PSs but to deliver them through microbial cells [203]. Liposomes, micelles, nanoemulsions are all used to self-assemble, mainly, the hydrophobic PSs once in an aqueous medium it can aggregate and decrease significantly the ROS produced [138,204].

Interestingly, the fullerenes (C60) are nanostructures that present PS properties such as a large molar absorption coefficient in visible light and high triplet yields. These PS-nanoparticles may switch the photochemical mechanism according to the medium, in aqueous environments the fullerenes are very efficient to produce HO* (Type I) while in hydrophobic medium produce singlet oxygen (Type II) [138,205]. Generally, the C60 are derivatives with functional groups to be used as PS and to exhibit biological compatibility. According to the literature cationic fullerenes, are mostly aPDT efficient against various classes of microbial cells [206]. The di-serinol-functionalized C_60_ presents to be more promisor once presents no dark toxicity, but shows a typical light dose-dependent loss of colony-forming ability [207].

As commented above the phenalenones are also an important PS used in aPDT studies. Despite this BODIPY dyes can be negatively or positively charged PS, and exhibit high singlet oxygen quantum yields, acting almost always accord to Type II mechanism [126].

Within the background, it is possible to assume that at this moment there is not an ideal PS developed to successfully disrupt 100% of microbial biofilms. Therefore, it is important to combine the aPDT with an approach/technique that enables, at the same time, the bind of the PS to EPS and its permeabilization through the extracellular matrix to reach deeper cells of the biofilm, enabling efficiently the ROS production and action. The PEF is an applicable example of that once can form porous in the cells membrane and through biofilm [208], can also stimulate the ROS production [16].

#### Light sources

The light source is essential for aPDT once is the one that activates the PS to produce ROS. Some basics requirements have been made for aPDT light source, including their electronic absorption spectrum that should match with PS activation spectrum (usually the longest wavelength peak), they need to be deliverable to the target tissue ergonomically and with high efficiency, must be reliable in the clinical environment and be cost-effective [209].

In addition, it is necessary to consider body tissues as a bulk medium, where the light propagation can suffer the processes of refraction, reflection, absorption and scattering [135]. Refraction that is governed by Snell’s law [210] and reflection from the interface between two media governed by Fresnel’s law [211] show processes that are proportional to the angle of incidence; i.e one can minimize them by applying the light beam perpendicular to the interface between the two media [135,212]. According to [213], reflection and refraction process impacts on the loss of intensity are determined by the relative values of their refractive indices. Light scattering in tissue has a pronounced effect on light intensity and directionality. When scattering occurs together with refraction, results in a loss of fluence rate (given as power per unit area of light in [W.m^−2^]) as well as a change in the directionality of the light beam, due a widening of the light beam [214].

Absorption depends on tissue composition, it is determined by the aPDT “optical window” and can be minimized by activation in the far-red wavelength region [135,212]. In tissue, the main chromophores are water, oxyhemoglobin (HbO_2_) and deoxyhemoglobin, melanin and cytochromes [215], and their absorption spectra define the optical window for aPDT [137]. It is known that hemoglobin (Hb) and HbO_2_ presents absorption in the range of 600–800 nm, and in some cases (e.g. in dental caries) hemoglobin chromophore may be absent and consequently, blue and green light would present no interference-effect [135].

Thus, most PS’s are activated by red light between 630 and 700 nm, corresponding to a light penetration depth from 0.5 cm to 1.5 cm, limiting to reach the depth of necrosis [216]. The light source, total light dose, dose rates and aPDT effect depend on the localization of the tissue treated and the PS applied [217].

The first light source used in aPDT was the conventional bulb, which did not yield good results once presented properties as polychromaticity, strong thermal component, and incoherency [218]. Nowadays, three main classes of aPDT light sources are being applied: lasers (e.g. argon, diode, or neodymium doped: yttrium, aluminum, and garnet [Nd:YAG] lasers), light-emitting diodes (LEDs) and gas-discharge lamps (e.g. quartz-tungsten-halogen or xenon-discharge lamps) [96].

Lasers are very convenient and reliable, due to their monochromaticity, high efficiency (>90%) of coupling into single optical fibers and high potency, as well as they can be interstitial light delivery devices. However, they do have a high cost (being Diode laser one of the lowest-priced among them), has a single wavelength requiring a separate unit according to PS used [219].

On the other hand, LED presents advantages over lasers such as low cost and ease configuration that arrays into different irradiation geometries, they are more compact and portable [209,219,220]. The output power used is no longer a limitation for LED, once it arrays with hundreds of mWcm^−2^ are available spanning most of the visible–NIR spectrum. Thus, linear arrays of LEDs can be used endoscopically or even interstitially, but they continue presenting relatively poor electrical-to-light conversion efficiency of conventional LEDs [209]. LED output spectrum typically has a bandwidth of around 25 to 30 nm and an efficiency factor for the typical PS spectrum of about 50%, the same can be noted with filtered halogen lamps [209]. The main characteristic of filtered halogen lamps is that they can be spectrally filtered to match any PS, but they can cause heating and cannot be efficiently coupled into optical fiber bundles or liquid light guides [219]. In addition, with broadband sources, their effective output potency is lesser than lasers’ source but proportional to the integrated product of the source output spectrum and the PS activation spectrum [221].

An important point is about the irradiation period and the respective light source used, that depending on the energy dose applied, can cause a temperature increase, leading to tissue damages [96,222]. Have been reported that the dose of energy is a parameter that generally shows a great variation, correlated to different potencies and exposure times. The highest potencies are related to lasers sources [170], once they can concentrate great energy in a small area, as tungsten lamps that adopted longer irradiation time once present a broad spectrum and low intensity requiring longer time interval to achieve an efficient energy emission [219,223].

Concerning continuous light and pulsed light sources, there are still controversial points of view and no conclusions. However, for biofilms infection, we may highlight pulsed light for two special reasons: (i) it allows recovery of the level of regular oxygen; (ii) allows the temperature of tissue to be kept at acceptable levels [224].

Therefore [209], suggested that for high aPDT efficiency, it is required: the presence of more regular oxygen close to the PS and the target tissue, multi-wavelength laser diode systems and LED arrays with user-configurable geometry to match with the treatment area, development of disposable packages with an integrated light source and delivery components, ultrafast (fs) pulsed laser sources for two-photon; and all that with low-cost systems, mainly for treating localized infections.

### Pulsed electric field

#### Fundamental mechanism

The plasma membrane of the cell could be imagined as an insulator layer with an aqueous solution of electrolyte at both sides. When cells are exposed to high power external pulsed field, cell membrane permeability to various impermeable molecules increases suddenly, this process is called electroporation or electropermeabilization. It is the most revealed bioelectric effect on the cells. Then, the pulsed electric field polarizes (rotate) the water and other dipolar molecules, e.g., lipid head groups, this can modulate the transmembrane potential and locally can reach a critical permeabilizing threshold (0.2–0.6 V) by inducing some local disorders (pores) in a lipid membrane [225]. There are many excellent reviews devoted to electroporation and membrane permeabilization in general [226,227]. If the electric field parameters are above some certain level, cells are not able to return to an intact state and the process thus leads to cell death. However, membranes of living cells can spontaneously return to their primal state if the electric field is not too intense. In classical cell membrane electropermeabilization the electric field pulse lasts from submicro- to several milliseconds and does not exceed 10 kV/cm electric field strength for eukaryotic cells [228]. In reference to the pulse length, they could be divided into three main categories: mili-, micro- and nano-second length pulses each of them having a different bioelectric effect on living cells. Basically, the characteristics of electric field parameters for various targets differ depending on the final aim of the PEF treatment, experimental configuration, evaluation methods, the characteristics of the electroporation buffer, cell culture or tissue. In case the cells *in vitro* must be destroyed, electric field parameters are chosen above some certain level, where the cells can’t return to the intact state and die. Meanwhile, there is no evidence of PEF application on biofilms in clinics. The second bioelectric effect on the cells is the generation of ROS after exposure to PEF. It can be (i) direct when free radicals are formed at the surface of the electrodes and (ii) indirect when free radicals are generated as a cell reaction to the PEF as abiotic stress. Both types are further discussed.

#### PEF effect to the cells and biofilm

Interestingly, the generation of oxygen reactive species comparing the cells exposed to micro- and milliseconds range electric field strength was more significant in the samples treated with milliseconds pulsed electric field (mPEF). After such treatment, the mammalian cell membrane was permeabilized reversibly. It was concluded, that increased production of ROS was directly associated with a permeabilized state of the plasma membrane [229]. Such ROS generation might be induced by the oxidative stress associated with the local nonspecific permeabilization of the plasma membrane causing the peroxidation of the lipids (Table 3) [230]. However, there is some contradictory experimental evidence with the yeast and bacteria cells, where ROS generation was not associated with plasma membrane permeability. The data support the idea that intracellular ROS generation by the mPEF is determined by the metabolic activity of the cells [230,231]. There is no correlation between permeabilization and ROS generation when short (microsecond) pulsed electric field (µPEF) was applied [232]. Furthermore, experimental studies have shown that even shorter sub-nanosecond and nanosecond pulsed electric field (nsPEF) can generate intracellular and extracellular ROS and it might be involved in lipids oxidation, but do not associate with plasma membrane permeabilization [233,234]. Theses experimental evidence support the idea that the mechanisms of ROS generation by the short electrical pulses (nsPEF) are fundamentally different from which causes an increase in ROS generation by the longer (mPEF) pulses.

**Table 3.** The ROS type generated after treatment with PEF in various types of cells

Electric field parameters	ROS type	Cells	References
0.5 kV/cm; 1 Hz; 6 ms 0.9 kV/cm; 1 Hz; 10–100 μs	H_2_O_2_-	Chinese hamster ovary (CHO)	[229]
5 kv/cm; 25 μF, 200 Ω; 600 μs	Hydroperoxides	Erythroleukemia K562	[230]
1.75 to 3.25 kV/cm; pulse number up to 9; up to 1 ms	General ROS	Saccharomyces cerevisiae	[231]
0.44 kV/cm; 1 Hz; 1 ms 0.44 kV/cm; 1 Hz; 100 μs	General ROS-	CHO	[232]
1–13 kV/cm; 1 Hz; 300 ns	H_2_O_2_	Jurkat	[234]
30 kV/cm; pulse number up to 10; 100 ns	General ROS	Homo sapiens pancreas BxPC-3 cells	[242]

Therefore, the increased ROS production in permeabilized cell by µPEF remain unclear. One of the possible explanations could be that the source of ROS generation might be a membrane NADPH-dependent oxidase (NOX) [235]. The calcium depended NOX activation is very important for cellular defense against viruses and other biotic and abiotic stresses [236]. Moreover, µPEF, as abiotic stress, involved in intracellular calcium release might be an indirect activator of NOX. It is hard to speculate because there is indirect evidence that NADPH could be released from the yeast or mammalian cells after permeabilization too [237–239]. Unfortunately, there is no direct evidence that activation of NOX depends on pulsed electric field treatment of eukaryotic cells.

Electrical pulses of durations between 10 and 1000 ns (nsPEF) usually affect intracellular structures and create pores in the cell plasma membranes (Table 3) [240,241]. Electropermeabilizing pulses of 100 ns long and 30 kV/cm strength triggered an increase in reactive oxygen species (ROS) generation in human pancreatic carcinoma cells and such a generation of ROS can be downregulated by modulating an intracellular Ca^2+^ concentration by adding an antioxidant [Nuccitel^242^]. It was concluded, that Ca^2+^ is an important effector for ROS generation by nsPEF. Moreover, it was shown, that nsPEF pulses with moderate duration (τ = 10–90 ns) induce caspase-dependent apoptosis in yeast cells and most likely such a programmed cell death process could be triggered by cytochrome C release from nsPEF permeabilized mitochondria in the presence of Ca^2+^ [243,244]. Such hypothesis can be justified considering that ROS is derived from mitochondria after mitochondrial permeability transition pore formation. Finally, ROS is an important player which facilitates apoptosome formation in eukaryotic cells [245]. Furthermore, present evidence shows that nsPEFs generate mitochondrial ROS that activate heme-regulated inhibitors, leading to eIF2α phosphorylation by modulating eukaryotic integrated stress response [246].

The second way of ROS formation is the direct burst of ROS at the surface of the electrodes during the PEF treatment [247]. The extent of ROS formation also depends on the electrode material [248,249]. During PEF treatment cell suspension is exposed to a strong electric current that passes the solution. Various electrochemical reactions occur at the interface of electrode–solutions, electrons are transferred from or to an electrode and the charge is thus allowed to flow completely around the circuit. At each electrode–solution interface, electrochemical half-reactions of the species with the most favorable redox potential take place, namely reduction on the cathode and oxidation on the anode. Cathodic reactions include the reduction of metal cations, reduction of water. Anodic reactions include oxidation of water, anions and metals [247]. Besides primary electrochemical reactions happening at the electrode–solution interface, the secondary chemical reactions might also appear including those, generating ROS formation:

(2) $$Cl_{2}+H_{2}O\Leftrightarrow HClO+H^{+}+Cl^{-}$$

(3a) $$Fe^{2+}+H_{2}O_{2}\to Fe^{3+}{+}^{\;}OH+OH^{-}$$

(3b) $$Fe^{3+}+H_{2}O_{2}\to Fe^{2+}{+}^{\;}OOH+H^{+}$$

(4) $$O_{2}+2H^{+}+2e^{-}\to H_{2}O_{2}$$

Longer, microsecond range electrical pulses lead to electrolysis and changes in pH and thus the generation of free radicals [248,249]. The electrode material also matters for the quantity of ROS generated at the electrode surface. Higher electropermeabilization efficacy comes with higher ROS formation, especially when copper electrodes are used in comparison to aluminum and steel electrodes applying both nanosecond and microsecond pulses range. However, not only the pH changes but also metal ion release proved to play a significant role in ROS formation [248,249].

For example, the formation of H_2_O_2_ was proposed to happen through nsPEF electrochemical effect on the electrodes. Very high intensity is associated to a short-lived current. The increased oxidation of Amplex Red (N-Acetyl-3,7-dihydroxyphenoxazine) occurred due to the formation of H_2_O_2_ in nsPEF-treated media, specifically, H_2_O_2_ in the presence of horseradish peroxidase (HRP) promotes high fluorescent resorufin (fluorescence). On the other hand, the ROS generation by the buffer was not reported (e.g. longer pulses as usual used in electroporation. nsPEFs generated both extracellular (in part electrochemical) and intracellular ROS, among which H_2_O_2_ [250].

One of the conclusions summarized in this part of the review was that nsPEFs were the most likely to cause most intracellular effects leading the ROS production. It is clear, that most of these evidence were observed in mammalian cells. More experimental results on nsPEF-induced ROS production in eukaryotic microbes, like yeast, would be very profitable for developing fused technologies in the future.

Several studies have been reported the effect of PEF against several microbial biofilms [16,251]. applied 200 Hz pulsed electric field of 18 V/cm for 2.5 µsec and then sonicated per 5 minutes, reducing 95% of *S. aureus* attachment. On the other hand [252], applied low-voltage (0.5–5 V) pulsed electric fields using an interdigitated electrodes with 29 μm spacing between 22-μm-wide electrodes to prevent *P. aeruginosa* biofilm development [253]. also investigated the effect of PEF against *P. aeruginosa*, applying 1500 V through a central electrode, with pulse duration of 50 ms, and pulse delivery frequency of 2 Hz. Bioluminescent imaging and Scanning Electron Microscopy showed that the area at which 100–80% of bacteria were eradicated was 50.5 ± 9.9 mm^2^ for 300 pulses, and 13.4 ± 0.65 mm^2^ for 150 pulses. In addition [254], proposed a new methodology centered on nanosecond high frequency electric field, which can successfully eradicate bacteria in *in vivo* studies models. They applied a high frequency 15kV/cm, with up to 900 ns pulsing bursts in combination with acetic acid (0.1–1%) to treat *P. aeruginosa* in a murine model. With the results, they suggested a direct application of PEF for treatment of wounds and ulcers when chemical treatment is no longer effective.

These studies have contributed to the discussion about the mechanism of action of the bioelectric effect and some hypotheses were formulated as (I) reduction of the biofilm capacity for binding to the antimicrobial agent [255]; (II) electrophoretic augmentation of the antimicrobial agent transport [256]; (III) membrane permeabilization [256]; (IV) electrolytic generation of oxygen [257]; (V) electrochemical generation of potentiating oxidants [258]; (VI) cell wall and membrane permeabilization [259].

## Future research directions for the combination of aPDT and PEF, against microbial biofilm

aPDT system should present characteristics to be effective to disrupt and kill the biofilm cells, without any side-effects for human body [96]. It has been reported that these characteristics are, mainly, head to PS permeabilization and it ROS production. Thus, an ideal PS must present characteristics as aqueous medium affinity and presence of positive charges [260], low molecular weight [96], high photostability [261] and singlet oxygen yield [140], and no toxicity or mutagenicity [262]. However, within the background, it is possible to verify that until now there is not any class of the dye showing these all characteristics.

Thus, alternatives have been proposed to enhance one or more of the PS properties. In this review, we are suggesting that a merge of aPDT to PEF may promote the increase of PS permeabilization, even hydrophobic ones as well as enhance the amount of ROS produce, to be applied against biofilm. The PEF is not a powerful generator of ROS as aPDT, but [16] reported that this technique presents mechanisms as electrochemical generation of potentiating oxidants and an indirect mechanism to produce ROS as electrolytic generation of oxygen.

Currently, the combination of light with PEF to prevent or kill microorganisms has been shown [263]. conducted a study combining ultraviolet light (UV) irradiation, PEF, and ozone for the inactivation of *E. coli* O157:H7 in poultry chiller water. As result after the application of 200 pulses and an electric field at 30 kV/cm, occurred a reduction of 4.1 log_10_ of *E. coli*. Most of the studies about PEF and light are focusing on food preservation or decontamination. According to [264] the merge of non-thermal hurdles such as ultraviolet light (UV) (5.3 J/cm^2^), high-intensity light pulses (HILP) (3.3 J/cm^2^), PEF (34 kV/cm, 18 Hz, 93 μs) decreased 6.0 log of *E. coli* [265], studied the effect of PEF and UV against six microorganisms (*S. aureus, L. monocytogenes E. coli, Salmonella spp., Cronobacter sakazakii* and *Campylobacter jejuni*) and realized that only PEF was able to induce any damage to the microorganism, mainly in neutral pH. However, by changing the pH and applying light, the PEF microorganism resistance decreased.

Although the association of PEF and light demonstrated promising results against microorganisms, the idea was not very explored in aPDT field but applied in PDT for cancer treatment [266,267]. At this moment, only one paper reported the effect of aPDT and PEF association, against microorganisms. In this study, they evaluated the effect of aPDT with PEF on the planktonic cells *S. aureus* and *E. coli*. After irradiation (590 nm) of hydrophobic PS, hypericin, the microorganisms were inactivated in 2.65 logs and 3.67 logs more than aPDT alone, respectively. The authors concluded that the PEF improves the delivery of PS into the cells, enhancing the aPDT effect [15].

**Figure 6.**
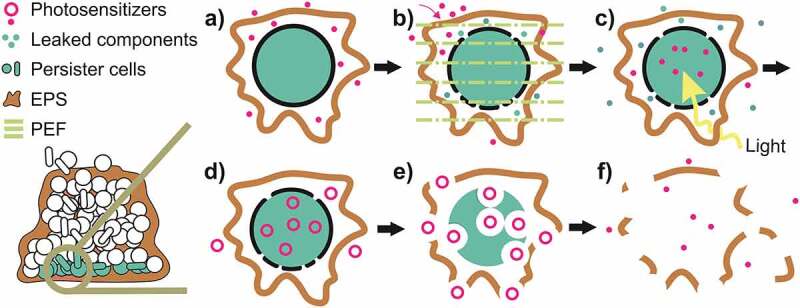
Hypothetical mechanism of action by the association of aPDT and PEF. **a)** Mature biofilm. **b)** *Persister cells* surrounded by EPS and the PS binding with the EPS and trying to reach the *persister cells*. **c)** Application of PEF, permeabilizing the PS diffusion through the EPS and cell membrane. **d)** Application of visible light, corresponding to the PS absorption. **e)** PS activation for ROS production. **f)** Production of ROS and its actions against both cells and EPS. **e)** The disruption of EPS, consequently also of the biofilm matrix and death of the microbial cell. PS returns to its ground state

So, the necessity for more studies to evaluate the influence of the PEF in the aPDT effect against microbial biofilm still stands. Despite that, we hypothesize how the association of aPDT with PEF can act over the biofilm and increase microbial death (Figure 6). The PEF would allow the PS diffusion through the extracellular matrix, increasing the PS concentration to bind with the EPS and biofilm cells. In addition, the PS may be allocated in all biofilm layers and reach the deeper cells (*persisters* cells), preventing the biofilm re-grow. ROS production by PS will be not affected by PEF; on the contrary, it will contribute to increase their yield.

## Authors opinion

Given the many mechanisms of biofilm resistance, the permeability of the EPS is one of the most important issues, especially talking about the aPDT effectiveness. It is known that the biofilm porosities change from top layers to the bottom layers, while the biofilm mean pore radius decreases in the bottom layers. These aspects corroborate with the PS difficulty to access the deepest biofilm cells (dormant cells) decreasing the aPDT effect. Despite the ratios of viable cells to total biomass in the biofilm bottom layers (~39%) being around twice less than the cells in the top (~91%), deeper cells are one of the most important targets for the biofilm treatment. Since the bottom cells (dormant cells) have lower metabolism and are protected by EPS it became a challenge to find a way to reach and kill them. Therefore, electroporation could be a technique to overcome that, enhancing the biofilm permeability as well as increasing the amount of ROS produced. It is important to highlight the need to conduct more studies about the effect of aPDT and PEF against the QS molecules due to their importance for EPS production, cells virulence and pathogenicity, promoting biofilm development and resistance. It is also necessary to conduct thorough studies of the combination of two techniques in order two fully understand the benefits and to select the best parameters for biofilm treatment.

## Executive summary

### *Conditions that should be* taken in *for aPDT and PEF application*

- Understanding of photosensitizer chemical, physical and “biological” properties, once the clinician has to select a PS that seems most suitable for the patient’s needs (severity and location of infection) and with the best clinical features.
- PS should present 1) maximum absorption corresponding to the window between 650 nm and 850 nm, where tissue penetration is quite high; 2) energy for the triplet state that is sufficient for singlet oxygen production, 3) low photo-degradation that is optimal for extended irradiation times, 4) amphiphilicity to ensures both transportations in the blood and penetration through the lipid layer of the cell membrane, hinder it precipitation or aggregation, 5) selectivity to accumulate in the target (microbial cells) tissue but not in normal (healthy) cells, restricting photo-induced damage to surrounding tissue and 6) should not cause mutagenic effects, irrespective of being irradiated or not.
- The main aspect to choose a light source is the spectral emission (corresponding to PS wavelength absorption), intensity/power and mode of light-delivery (via optical fiber or directly) both suitable for the localization and severity of the infection.
- Careful attention to physics and dosimetry of aPDT will help to minimize general toxicity and side effects.
- Develop an ideal photosensitizer is an important goal for clinical application of aPDT to be effective, but not prevent the use of supplementary forms of treatment that can be combined with aPDT, as PEF.
- Microbial inactivation by PEF, mainly nanosecond PEF, is governed by the treatment time (number of pulses × duration of the pulse), knowing that microbial inactivation increases with an increase in the number of pulses. This approach could represent an alternative to aPDT, increasing and delivering the PS to the local of biofilm infection, special the ones that occur as wound. However, more fundamental evidence and technical developments are needed to understand the effects of PEF on biofilms and it synergetic effect in combination with aPDT.
- Define optimized and standard protocols of PEF/aPDT that can suitable for a particular application on clinical, according to the tissue localization (wound or medical device) and species of microorganisms (multiple or single species) of the biofilm infection.

## References

[1] World Health Organization‎. Antimicrobial resistance: global report on surveillance. World Health Organization. 2014. https://apps.who.int/iris/handle/10665/112642

[2] Smith R, Coast J. The true cost of antimicrobial resistance. Bmj. 2013;346:f1493. 23479660 10.1136/bmj.f1493

[3] England PH. Keep antibiotics working [Online]. British society for antimicrobial therapy;2020 [cited 2020 May 14].

[4] Davies D. Understanding biofilm resistance to antibacterial agents. Nat Rev Drug Discov. 2003;2(2):114–122. 12563302 10.1038/nrd1008

[5] Koo H, Allan RN, Howlin RP, et al. Targeting microbial biofilms: current and prospective therapeutic strategies. Nature Rev Microbiol. 2017;15(12):740–755. 28944770 10.1038/nrmicro.2017.99 PMC5685531

[6] Wu H, Moser C, Wang H-Z, et al. Strategies for combating bacterial biofilm infections. Int J Oral Sci. 2015;7(1):1–7. 25504208 10.1038/ijos.2014.65 PMC4817533

[7] de la Fuente-Núñez C, Reffuveille F, Fernández L, et al. Bacterial biofilm development as a multicellular adaptation: antibiotic resistance and new therapeutic strategies. Curr Opin Microbiol. 2013;16(5):580–589. 23880136 10.1016/j.mib.2013.06.013

[8] Ciofu O, Rojo-Molinero E, Macià MD, et al. Antibiotic treatment of biofilm infections. APMIS. 2017;125(4):304–319. 28407419 10.1111/apm.12673

[9] Lewis K. Persister cells, dormancy and infectious disease. Nature Rev Microbiol. 2007;5(1):48–56. 17143318 10.1038/nrmicro1557

[10] Hamblin MR, Abrahamse H. Oxygen-independent antimicrobial photoinactivation: type III photochemical mechanism? Antibiotics (Basel). 2020;9:2. 10.3390/antibiotics9020053 PMC7168166 32023978

[11] Viens AM, Littmann J. Is antimicrobial resistance a slowly emerging disaster? Public Health Ethics. 2015;8(3):255–265. 26566396 10.1093/phe/phv015 PMC4638061

[12] Dryden MS, Cooke J, Salib RJ, et al. Reactive oxygen: a novel antimicrobial mechanism for targeting biofilm-associated infection. J Glob Antimicrob Resist. 2017;8:186–191. 28213334 10.1016/j.jgar.2016.12.006

[13] Rajesh S, Koshi E, Philip K, et al. Antimicrobial photodynamic therapy: an overview. J Indian Soc Periodontol. 2011a;15(4):323–327. 22368354 10.4103/0972-124X.92563 PMC3283927

[14] Wainwright M. Photodynamic antimicrobial chemotherapy (PACT). J Antimicrob Chemother. 1998;42(1):13–28. 9700525 10.1093/jac/42.1.13

[15] de Melo WDCMA, Lee AN, Perussi JR, et al. Electroporation enhances antimicrobial photodynamic therapy mediated by the hydrophobic photosensitizer, hypericin. Photodiagnosis Photodyn Ther. 2013b;10(4):647–650. 24284122 10.1016/j.pdpdt.2013.08.001 PMC3889204

[16] Del Pozo JL, Rouse MS, Patel R. Bioelectric effect and bacterial biofilms. A systematic review. Int J Artif Organs. 2008;31(9):786–795. 18924090 10.1177/039139880803100906 PMC3910516

[17] López D, Vlamakis H, Kolter R. Biofilms. Cold Spring Harb Perspect Biol. 2010;2(7):a000398–a000398. 20519345 10.1101/cshperspect.a000398 PMC2890205

[18] Bryers JD. Medical biofilms. Biotechnol Bioeng. 2008;100(1):1–18. 18366134 10.1002/bit.21838 PMC2706312

[19] Donlan RM. Biofilms: microbial life on surfaces. Emerg Infect Dis. 2002;8(9):881–890. 12194761 10.3201/eid0809.020063 PMC2732559

[20] Del Pozo JL. Biofilm-related disease. Expert Rev Anti Infect Ther. 2018;16(1):51–65. 29235402 10.1080/14787210.2018.1417036

[21] Francolini I, Donelli G. Prevention and control of biofilm-based medical-device-related infections. FEMS Immunol Med Microbiol. 2010;59(3):227–238. 20412300 10.1111/j.1574-695X.2010.00665.x

[22] Mack D, Rohde H, Harris LG, et al. Biofilm formation in medical device-related infection. Int J Artif Organs. 2006;29(4):343–359. 16705603 10.1177/039139880602900404

[23] Sandoe JAT, Witherden IR, Cove JH, et al. Correlation between enterococcal biofilm formation in vitro and medical-device-related infection potential in vivo. J Med Microbiol. 2003;52(7):547–550. 12808074 10.1099/jmm.0.05201-0

[24] Stewart PS, Bjarnsholt T. Risk factors for chronic biofilm-related infection associated with implanted medical devices. Clin Microbiol Infect. 2020;26(8):1034–1038. 32120041 10.1016/j.cmi.2020.02.027

[25] Carmona-Torre F, Yuste JR, Castejon S, et al. Catheter-related bloodstream infections in patients with oncohaematological malignancies. Lancet Infect Dis. 2017;17(2):139–140. 28134110 10.1016/S1473-3099(17)30011-7

[26] Andersson DI, Hughes D. Microbiological effects of sublethal levels of antibiotics. Nature Rev Microbiol. 2014;12(7):465–478. 24861036 10.1038/nrmicro3270

[27] Gupta P, Sarkar S, Das B, et al. Biofilm, pathogenesis and prevention–a journey to break the wall: a review. Arch Microbiol. 2016;198(1):1–15. 26377585 10.1007/s00203-015-1148-6

[28] Rinaudi L, Giordano W. An integrated view of biofilm formation in rhizobia. FEMS Microbiol Lett. 2009;304:1–11. 19930462 10.1111/j.1574-6968.2009.01840.x

[29] Delattin N, Cammue BP, Thevissen K. Reactive oxygen species-inducing antifungal agents and their activity against fungal biofilms. Future Med Chem. 2014;6(1):77–90. 24358949 10.4155/fmc.13.189

[30] Ramage G, Rajendran R, Sherry L, et al. Fungal biofilm resistance. Int J Microbiol. 2012;(2012:528521. 22518145 10.1155/2012/528521 PMC3299327

[31] Flemming HC, Wingender J. The biofilm matrix. Nat Rev Microbiol. 2010;8(9):623–633. 20676145 10.1038/nrmicro2415

[32] Hall-Stoodley L, Stoodley P. Evolving concepts in biofilm infections. Cell Microbiol. 2009;11:1034–1043. 19374653 10.1111/j.1462-5822.2009.01323.x

[33] Melo WCMA, Perussi JR. Strategies to overcome biofilm resistance. In: Méndez-Vilas. A, editor. Microbial pathogens and strategies for combating them: science, technology and education. Badajoz, Spain: Formatex Research Center; 2013. p. 179–187.

[34] Jamal M, Ahmad W, Andleeb S, et al. Bacterial biofilm and associated infections. J Chin Med Assoc. 2018;81(1):7–11. 29042186 10.1016/j.jcma.2017.07.012

[35] Kostakioti M, Hadjifrangiskou M, Hultgren SJ. Bacterial biofilms: development, dispersal, and therapeutic strategies in the dawn of the postantibiotic era. Cold Spring Harb Perspect Med. 2013;3(4):a010306–a010306. 23545571 10.1101/cshperspect.a010306 PMC3683961

[36] de Melo WCMA, Avci P, de Oliveira MN, et al. Photodynamic inactivation of biofilm: taking a lightly colored approach to stubborn infection. Expert Rev Anti Infect Ther. 2013a;11(7):669–693. 23879608 10.1586/14787210.2013.811861 PMC4336791

[37] Donlan RM. Biofilm formation: a clinically relevant microbiological process. Clinl Infect Dis. 2001;33(8):1387–1392. 10.1086/322972 11565080

[38] Wood TK, Knabel SJ, Kwan BW. Bacterial persister cell formation and dormancy. Appl Environ Microbiol. 2013;79(23):7116–7121. 24038684 10.1128/AEM.02636-13 PMC3837759

[39] Lewis K. Persister Cells. Annu Rev Microbiol. 2010;64(1):357–372. 20528688 10.1146/annurev.micro.112408.134306

[40] Solano C, Echeverz M, Lasa I. Biofilm dispersion and quorum sensing. Curr Opin Microbiol. 2014;18:96–104. 24657330 10.1016/j.mib.2014.02.008

[41] Rutherford ST, Bassler BL. Bacterial quorum sensing: its role in virulence and possibilities for its control. Cold Spring Harb Perspect Med. 2012;2(11):a012427. 23125205 10.1101/cshperspect.a012427 PMC3543102

[42] Pérez-Pérez M, Jorge P, Pérez Rodríguez G, et al. Quorum sensing inhibition in Pseudomonas aeruginosa biofilms: new insights through network mining. Biofouling. 2017;33(2):128–142. 28121162 10.1080/08927014.2016.1272104

[43] Waters C, Bassler B. Quorum sensing: cell-to-cell communication in bacteria. Annu Rev Cell Dev Biol. 2005;21:319–346. 16212498 10.1146/annurev.cellbio.21.012704.131001

[44] Yin W-F, Tung H-J, Sam C-K, et al. Quorum quenching Bacillus sonorensis isolated from soya sauce fermentation brine. Sensors (Basel). 2012;12(4):4065–4073. 22666018 10.3390/s120404065 PMC3355399

[45] Novick RP, Projan SJ, Kornblum J, et al. The agr P2 operon: an autocatalytic sensory transduction system in Staphylococcus aureus. Mol Gen Genet. 1995;248(4):446–458. 7565609 10.1007/BF02191645

[46] Seed PC, Passador L, Iglewski BH. Activation of the Pseudomonas aeruginosa lasI gene by LasR and the Pseudomonas autoinducer PAI: an autoinduction regulatory hierarchy. J Bacteriol. 1995;177(3):654–659. 7836299 10.1128/jb.177.3.654-659.1995 PMC176640

[47] Kaplan HB, Greenberg EP. Diffusion of autoinducer is involved in regulation of the Vibrio fischeri luminescence system. J Bacteriol. 1985;163(3):1210–1214. 3897188 10.1128/jb.163.3.1210-1214.1985 PMC219261

[48] Hooshangi S, Bentley W. From unicellular properties to multicellular behavior: bacteria quorum sensing circuitry and applications. Curr Opin Biotechnol. 2008;19:550–555. 18977301 10.1016/j.copbio.2008.10.007

[49] Kalia VC, Purohit HJ. Quenching the quorum sensing system: potential antibacterial drug targets. Crit Rev Microbiol. 2011;37(2):121–140. 21271798 10.3109/1040841X.2010.532479

[50] Ng WL, Bassler BL. Bacterial quorum-sensing network architectures. Annu Rev Genet. 2009;43:197–222. 19686078 10.1146/annurev-genet-102108-134304 PMC4313539

[51] Williams P, Cámara M. Quorum sensing and environmental adaptation in Pseudomonas aeruginosa: a tale of regulatory networks and multifunctional signal molecules. Curr Opin Microbiol. 2009;12(2):182–191. 19249239 10.1016/j.mib.2009.01.005

[52] Wang S, Shi W, Tang T, et al. Function of quorum sensing and cell signaling in the formation of aerobic granular sludge. Rev Environ Sci Bio/Technol. 2017;16(1):1–13.

[53] Sutherland I. Biofilm exopolysaccharides: a strong and sticky framework. Microbiology. 2001;147(Pt 1):3–9. 11160795 10.1099/00221287-147-1-3

[54] Zhou X, Meng J, Yu Z, et al. The alterations of biofilm formation and EPS characteristics of a diatom by a sponge-associated bacterium *psychrobacter* sp. Scientifica (Cairo). 2018;(2018:1892520. 30034907 10.1155/2018/1892520 PMC6035847

[55] Fulaz S, Vitale S, Quinn L, et al. Nanoparticle–biofilm interactions: the role of the EPS matrix. Trends Microbiol. 2019;27(11):915–926. 31420126 10.1016/j.tim.2019.07.004

[56] O’Toole G, Kaplan HB, Kolter R. Biofilm formation as microbial development. Annu Rev Microbiol. 2000;54:49–79. 11018124 10.1146/annurev.micro.54.1.49

[57] Shen Y, Stojicic S, Haapasalo M. Antimicrobial efficacy of chlorhexidine against bacteria in biofilms at different stages of development. J Endod. 2011;37(5):657–661. 21496666 10.1016/j.joen.2011.02.007

[58] Ferrer-Espada R, Liu X, Goh XS, et al. Antimicrobial blue light inactivation of polymicrobial biofilms. Front Microbiol. 2019a;10:721. 31024499 10.3389/fmicb.2019.00721 PMC6467927

[59] Gabrilska RA, Rumbaugh KP. Biofilm models of polymicrobial infection. Future Microbiol. 2015;10(12):1997–2015. 26592098 10.2217/fmb.15.109 PMC4944397

[60] Wolcott R, Costerton JW, Raoult D, et al. The polymicrobial nature of biofilm infection. Clin Microbiol Infect. 2013;19(2):107–112. 22925473 10.1111/j.1469-0691.2012.04001.x

[61] Ehrlich GD, Hu FZ, Shen K, et al. Bacterial plurality as a general mechanism driving persistence in chronic infections. Clin Orthop Relat Res. 2005;437(20–24). DOI:10.1097/00003086-200508000-00005 PMC1351326 16056021

[62] Peters BM, Jabra-Rizk MA, O’May GA, et al. Polymicrobial interactions: impact on pathogenesis and human disease. Clin Microbiol Rev. 2012a;25(1):193–213. 22232376 10.1128/CMR.00013-11 PMC3255964

[63] Burmølle M, Webb JS, Rao D, et al. Enhanced biofilm formation and increased resistance to antimicrobial agents and bacterial invasion are caused by synergistic interactions in multispecies biofilms. Appl Environ Microbiol. 2006;72(6):3916–3923. 16751497 10.1128/AEM.03022-05 PMC1489630

[64] Dalton T, Dowd SE, Wolcott RD, et al. An In Vivo polymicrobial biofilm wound infection model to study interspecies interactions. PLOS ONE. 2011;6(11):e27317. 22076151 10.1371/journal.pone.0027317 PMC3208625

[65] Ramsey MM, Rumbaugh KP, Whiteley M. Metabolite cross-feeding enhances virulence in a model polymicrobial infection. PLoS Pathog. 2011;7(3):e1002012. 21483753 10.1371/journal.ppat.1002012 PMC3069116

[66] Orazi G, O’Toole GA. “It takes a village”: mechanisms underlying antimicrobial recalcitrance of polymicrobial biofilms. J Bacteriol. 2019;202(1):e00530–00519. 31548277 10.1128/JB.00530-19 PMC6932244

[67] Peters BM, Jabra-Rizk MA, O’May GA, et al. Polymicrobial interactions: impact on pathogenesis and human disease. Clin Microbiol Rev. 2012b;25(1):193–213. 22232376 10.1128/CMR.00013-11 PMC3255964

[68] Rendueles O, Ghigo JM. Multi-species biofilms: how to avoid unfriendly neighbors. FEMS Microbiol Rev. 2012;36(5):972–989. 22273363 10.1111/j.1574-6976.2012.00328.x

[69] Lee KWK, Periasamy S, Mukherjee M, et al. Biofilm development and enhanced stress resistance of a model, mixed-species community biofilm. ISME J. 2014;8(4):894–907. 24152718 10.1038/ismej.2013.194 PMC3960537

[70] Ferrer-Espada R, Liu X, Goh XS, et al. Antimicrobial blue light inactivation of polymicrobial biofilms. Front Microbiol. 2019b;10:721-721. 10.3389/fmicb.2019.00721 PMC6467927 31024499

[71] Fourie R, Ells R, Swart CW, et al. Candida albicans and pseudomonas aeruginosa interaction, with focus on the role of eicosanoids. Front Physiol. 2016;7:64-64. 10.3389/fphys.2016.00064 PMC4767902 26955357

[72] Peleg AY, Hogan DA, Mylonakis E. Medically important bacterial–fungal interactions. Nature Rev Microbiol. 2010;8(5):340–349. 20348933 10.1038/nrmicro2313

[73] Trejo-Hernández A, Andrade-Domínguez A, Hernández M, et al. Interspecies competition triggers virulence and mutability in Candida albicans–Pseudomonas aeruginosa mixed biofilms. ISME J. 2014;8(10):1974–1988. 24739628 10.1038/ismej.2014.53 PMC4184018

[74] DeLeon S, Clinton A, Fowler H, et al. Synergistic interactions of pseudomonas aeruginosa and staphylococcus aureus in an in vitro wound model. Infect Immun. 2014;82(11):4718–4728. 25156721 10.1128/IAI.02198-14 PMC4249327

[75] Gjødsbøl K, Christensen JJ, Karlsmark T, et al. Multiple bacterial species reside in chronic wounds: a longitudinal study. Int Wound J. 2006;3(3):225–231. 16984578 10.1111/j.1742-481X.2006.00159.x PMC7951738

[76] Orazi G, Ruoff KL, O’Toole GA. Pseudomonas aeruginosa increases the sensitivity of biofilm-grown staphylococcus aureus to membrane-targeting antiseptics and antibiotics. mBio. 2019;10(4):e01501–01519. 31363032 10.1128/mBio.01501-19 PMC6667622

[77] Orazi G, O’Toole GA. Pseudomonas aeruginosa alters staphylococcus aureus sensitivity to vancomycin in a biofilm model of cystic fibrosis infection. mBio. 2017;8(4):e00873–00817. 28720732 10.1128/mBio.00873-17 PMC5516255

[78] Malhotra R, Dhawan B, Garg B, et al. A comparison of bacterial adhesion and biofilm formation on commonly used orthopaedic metal implant materials: an in vitro study. Indian J Orthop. 2019;53(1):148–153. 30905995 10.4103/ortho.IJOrtho_66_18 PMC6394199

[79] Freebairn D, Linton D, Harkin-Jones Freng E, et al. Electrical methods of controlling bacterial adhesion and biofilm on device surfaces. Expert Rev Med Devices. 2013;10:85–103. 23278226 10.1586/erd.12.70

[80] Erriu M, Blus C, Szmukler-Moncler S, et al. Microbial biofilm modulation by ultrasound: current concepts and controversies. Ultrason Sonochem. 2014;21(1):15–22. 23751458 10.1016/j.ultsonch.2013.05.011

[81] Qayyum S, Khan AU. Nanoparticles vs. biofilms: a battle against another paradigm of antibiotic resistance. MedChemComm. 2016;7(8):1479–1498.

[82] Rukavina Z, Vanić Ž. Current trends in development of liposomes for targeting bacterial biofilms. Pharmaceutics. 2016;8(2):18. 10.3390/pharmaceutics8020018 PMC4932481 27231933

[83] Tamilvanan S, Venkateshan N, Ludwig A. The potential of lipid- and polymer-based drug delivery carriers for eradicating biofilm consortia on device-related nosocomial infections. J Control Release. 2008;128(1):2–22. 18342974 10.1016/j.jconrel.2008.01.006

[84] Murthy, P.S., Venugopalan, V.P, Arunya, D.D, et al. Antibiofilm activity of nano sized CuO. International Conference on Nanoscience, Engineering and Technology. 2011;580–583. doi:10.1109/ICONSET.2011.6168037

[85] De Prijck K, De Smet N, Rymarczyk-Machal M, et al. (2010). Candida albicans biofilm formation on peptide functionalized polydimethylsiloxane. BIOFOULING26(3), 269–275. doi:10.1080/08927010903501908 20054722

[86] Sawada Si, Yamaguchi D, Putra A et al. Nanoscale structures of radiation-grafted polymer electrolyte membranes investigated via a small-angle neutron scattering technique. Polym J. 2013;45:797–801. doi:10.1038/pj.2012.218

[87] Kim, S.G., Yoon, Y.H., Choi, J.W., et al. Effect of furanone on experimentally induced Pseudomonas aeruginosa biofilm formation: in vitro study. Int J Pediatr Otorhinolaryngol. 2012;76(11):1575–8. doi:10.1016/j.ijporl.2012.07.015 22884365

[88] Giacometti A, Cirioni O, Gov Y, et al. RNA III inhibiting peptide inhibits in vivo biofilm formation by drug-resistant Staphylococcus aureus. Antimicrob Agents Chemother 2003;247(6):1979–83. doi:10.1128/AAC.47.6.1979-1983.2003 PMC155823 12760879

[89] Anguita-Alonso P, Giacometti A, Cirioni O, et al. RNAIII-inhibiting-peptide-loaded polymethylmethacrylate prevents in vivo Staphylococcus aureus biofilm formation. Antimicrob Agents Chemother. 2007;51(7):2594–6. doi:10.1128/AAC.00580-06 PMC1913233 17116671

[90] Wang J, Wen K, Liu X, Weng CX, Wang R, Cai Y. Multiple Low Frequency Ultrasound Enhances Bactericidal Activity of Vancomycin against Methicillin-Resistant Staphylococcus aureus Biofilms. Biomed Res Int. 2018:6023101. doi:10.1155/2018/6023101 PMC6186328 30364019

[91] Hou Y, Yang M, Jiang H, et al. Effects of low-intensity and low-frequency ultrasound combined with tobramycin on biofilms of extended-spectrum beta-lactamases (ESBLs) Escherichia coli.FEMS Microbiology Letters. 2019;366(3):fnz026. doi:10.1093/femsle/fnz026 30715289

[92] Sharma K Pagedar Singh A. Antibiofilm Effect of DNase against Single and Mixed Species Biofilm. Foods. 2018;7(3):42. doi:10.3390/foods7030042 PMC5867557 29562719

[93] Gomes F, Rodrigues ME, Martins N, et al. Phenolic Plant Extracts Versus Penicillin G: In Vitro Susceptibility of Staphylococcus aureus Isolated from Bovine Mastitis. Pharmaceuticals. 2019;12:128. doi:10.3390/ph12030128 PMC6789528 31480446

[94] Sánchez E, Morales CR, Castillo S, et al. Anibacterial and Antibiofilm Activity of Methanolic Plant Extracts against Nosocomial Microorganisms, Evidence-Based Complementary and Alternative Medicine. 2016. doi:10.1155/2016/1572697 PMC4939345 27429633

[95] Taraszkiewicz A, Fila G, Grinholc M, et al. Innovative strategies to overcome biofilm resistance. Biomed Res Int. 2013;(2013:150653. 23509680 10.1155/2013/150653 PMC3591221

[96] Cieplik F, Deng D, Crielaard W, et al. Antimicrobial photodynamic therapy - what we know and what we don’t. Crit Rev Microbiol. 2018;44(5):571–589. 29749263 10.1080/1040841X.2018.1467876

[97] Dai T, Huang YY, Sharma SK, et al. Topical antimicrobials for burn wound infections. Recent Pat Antiinfect Drug Discov. 2010;5(2):124–151. 20429870 10.2174/157489110791233522 PMC2935806

[98] Jori G, Fabris C, Soncin M, et al. Photodynamic therapy in the treatment of microbial infections: basic principles and perspective applications. Lasers Surg Med. 2006;38(5):468–481. doi: 10.1002/lsm.20361 16788934

[99] Hu X, Huang-Y-Y, Wang Y, et al. Antimicrobial photodynamic therapy to control clinically relevant biofilm infections. Front Microbiol. 2018;9:1299-1299. 10.3389/fmicb.2018.01299 PMC6030385 29997579

[100] Lam M, Jou PC, Lattif AA, et al. Photodynamic therapy with Pc 4 induces apoptosis of candida albicans. Photochem Photobiol. 2011b;87(4):904–909. 21521233 10.1111/j.1751-1097.2011.00938.x PMC3139787

[101] Lopes D, Melo T, Santos N, et al. Evaluation of the interplay among the charge of porphyrinic photosensitizers, lipid oxidation and photoinactivation efficiency in Escherichia coli. J Photochem Photobiol B. 2014a;141:145–153. 25463662 10.1016/j.jphotobiol.2014.08.024

[102] Dosselli R, Millioni R, Puricelli L, et al. Molecular targets of antimicrobial photodynamic therapy identified by a proteomic approach. J Proteomics. 2012;77:329–343. 23000218 10.1016/j.jprot.2012.09.007

[103] Konopka K, Goslinski T. Photodynamic therapy in dentistry. J Dent Res. 2007;86(8):694–707. 17652195 10.1177/154405910708600803

[104] Beirão S, Fernandes S, Coelho J, et al. Photodynamic inactivation of bacterial and yeast biofilms with a cationic porphyrin. Photochem Photobiol. 2014;90(6):1387–1396. 25112506 10.1111/php.12331

[105] Astuti SD, Hafidiana RR, Abdurachman PAP, et al. The efficacy of photodynamic inactivation with laser diode on Staphylococcus aureus biofilm with various ages of biofilm. Infect Dis Rep. 2020;12(Suppl1):8736-8736. 10.4081/idr.2020.8736 PMC7447954 32874465

[106] Garcez AS, Ribeiro MS, Tegos GP, et al. Antimicrobial photodynamic therapy combined with conventional endodontic treatment to eliminate root canal biofilm infection. Lasers Surg Med. 2007;39(1):59–66. 17066481 10.1002/lsm.20415 PMC3071045

[107] Diogo P, Fernandes C, Caramelo F, et al. Antimicrobial photodynamic therapy against endodontic enterococcus faecalis and candida albicans mono and mixed biofilms in the presence of photosensitizers: a comparative study with classical endodontic irrigants. Front Microbiol. 2017;8:498. 28424663 10.3389/fmicb.2017.00498 PMC5371592

[108] Luke-Marshall NR, Hansen LA, Shafirstein G, et al. Antimicrobial photodynamic therapy with chlorin e6 is bactericidal against biofilms of the primary human otopathogens. mSphere. 2020;5(4):e00492–00420. 32669474 10.1128/mSphere.00492-20 PMC7364218

[109] Quishida CCC, Carmello JC, Mima EGDO, et al. Susceptibility of multispecies biofilm to photodynamic therapy using photodithazine®. Lasers Med Sci. 2015;30(2):685–694. 23912779 10.1007/s10103-013-1397-z

[110] Collins TL, Markus EA, Hassett DJ, et al. The effect of a cationic porphyrin on pseudomonas aeruginosa biofilms. Curr Microbiol. 2010;61(5):411–416. 20372908 10.1007/s00284-010-9629-y

[111] Gonzales FP, Felgenträger A, Bäumler W, et al. Fungicidal photodynamic effect of a twofold positively charged porphyrin against Candida albicans planktonic cells and biofilms. Future Microbiol. 2013;8(6):785–797. 23701333 10.2217/fmb.13.44

[112] Di Poto A, Sbarra MS, Provenza G, et al. The effect of photodynamic treatment combined with antibiotic action or host defence mechanisms on Staphylococcus aureus biofilms. Biomaterials. 2009;30(18):3158–3166. 19329182 10.1016/j.biomaterials.2009.02.038

[113] Carmello JC, Alves F, Basso FG, et al. Antimicrobial photodynamic therapy reduces adhesion capacity and biofilm formation of Candida albicans from induced oral candidiasis in mice. Photodiagnosis Photodyn Ther. 2019;27:402–407. 31284075 10.1016/j.pdpdt.2019.06.010

[114] Jordão CC, de Sousa V, Inêz Klein T, et al. Antimicrobial photodynamic therapy reduces gene expression of Candida albicans in biofilms. Photodiagnosis Photodyn Ther. 2020;31:101825. 32445962 10.1016/j.pdpdt.2020.101825

[115] Astuty SD, Suhariningsih BA, Astuti SD. The Efficacy of Photodynamic Inactivation of the Diode Laser in Inactivation of the Candida albicans Biofilms With Exogenous Photosensitizer of Papaya Leaf Chlorophyll. J Lasers Med Sci. 2019;10(3):215–224. doi:10.15171/jlms.2019.35 PMC6817803 31749949

[116] Prates RA, Kato IT, Ribeiro MS, et al. Influence of multidrug efflux systems on methylene blue-mediated photodynamic inactivation of Candida albicans. J Antimicrob Chemother. 2011;66(7):1525–1532. 21525022 10.1093/jac/dkr160 PMC3112030

[117] Tegos GP, Hamblin MR. Phenothiazinium antimicrobial photosensitizers are substrates of bacterial multidrug resistance pumps. Antimicrob Agents Chemother. 2006;50(1):196–203. 16377686 10.1128/AAC.50.1.196-203.2006 PMC1346798

[118] Tegos GP, Masago K, Aziz F, et al. Inhibitors of bacterial multidrug efflux pumps potentiate antimicrobial photoinactivation. Antimicrob Agents Chemother. 2008;52(9):3202–3209. 18474586 10.1128/AAC.00006-08 PMC2533468

[119] Biel MA, Sievert C, Usacheva M, et al. Antimicrobial photodynamic therapy treatment of chronic recurrent sinusitis biofilms. Int Forum Allergy Rhinol. 2011a;1(5):329–334. 22287461 10.1002/alr.20089 PMC3270367

[120] Biel MA, Sievert C, Usacheva M, et al. Reduction of endotracheal tube biofilms using antimicrobial photodynamic therapy. Lasers Surg Med. 2011c;43(7):586–590. 21987599 10.1002/lsm.21103 PMC3188855

[121] Nastri L, Donnarumma G, Porzio C, et al. Effects of toluidine blue-mediated photodynamic therapy on periopathogens and periodontal biofilm: in vitro evaluation. Int J Immunopathol Pharmacol. 2010;23(4):1125–1132. 21244761 10.1177/039463201002300416

[122] Mahmoudi H, Pourhajibagher M, Alikhani MY, et al. The effect of antimicrobial photodynamic therapy on the expression of biofilm associated genes in Staphylococcus aureus strains isolated from wound infections in burn patients. Photodiagnosis Photodyn Ther. 2019;25:406–413. 30684672 10.1016/j.pdpdt.2019.01.028

[123] Misba L, Zaidi S, Khan AU. Efficacy of photodynamic therapy against Streptococcus mutans biofilm: role of singlet oxygen. J Photochem Photobiol B. 2018;183:16–21. 29680469 10.1016/j.jphotobiol.2018.04.024

[124] Araújo NC, Fontana CR, Bagnato VS, et al. Photodynamic antimicrobial therapy of curcumin in biofilms and carious dentine. Lasers Med Sci. 2014;29(2):629–635. 23793414 10.1007/s10103-013-1369-3

[125] Pereira CA, Costa ACBP, Carreira CM, et al. Photodynamic inactivation of Streptococcus mutans and Streptococcus sanguinis biofilms in vitro. Lasers Med Sci. 2013;28(3):859–864. 22847685 10.1007/s10103-012-1175-3

[126] Cieplik F, Tabenski L, Buchalla W, et al. Antimicrobial photodynamic therapy for inactivation of biofilms formed by oral key pathogens. Front Microbiol. 2014;5:405-405. 10.3389/fmicb.2014.00405 PMC4130309 25161649

[127] Cieplik F, Späth A, Regensburger J, et al. Photodynamic biofilm inactivation by SAPYR—An exclusive singlet oxygen photosensitizer. Free Radic Biol Med. 2013;65:477–487. 23891675 10.1016/j.freeradbiomed.2013.07.031

[128] Cieplik F. Pummer A, Regensburger J, Hiller KA, Späth A, Tabenski L, Buchalla W, Maisch T. The impact of absorbed photons on antimicrobial photodynamic efficacy. Fron Microbiol. 2015;6:706. doi:10.3389/fmicb.2015.00706 PMC4502582 26236292

[129] Biel MA, Sievert C, Usacheva M, et al. Antimicrobial photodynamic therapy treatment of chronic recurrent sinusitis biofilms. Int Forum Allergy Rhinol. 2011b;1(5):329–334. 22287461 10.1002/alr.20089 PMC3270367

[130] Dovigo LN, Carmello JC, Carvalho MT, et al. Photodynamic inactivation of clinical isolates of Candida using photodithazine®. Biofouling. 2013a;29(9):1057–1067. 24025068 10.1080/08927014.2013.827668

[131] Li X, Guo H, Tian Q, et al. Effects of 5-aminolevulinic acid-mediated photodynamic therapy on antibiotic-resistant staphylococcal biofilm: an in vitro study. J Surg Res. 2013;184(2):1013–1021. 23622723 10.1016/j.jss.2013.03.094

[132] Bardaweel SK, Gul M, Alzweiri M, et al. Reactive oxygen species: the dual role in physiological and pathological conditions of the human body. Eurasian J Med. 2018;50(3):193–201. 30515042 10.5152/eurasianjmed.2018.17397 PMC6263229

[133] Milkovic L, Cipak Gasparovic A, Cindric M, et al. Short overview of ROS as cell function regulators and their implications in therapy concepts. Cells. 2019;8(8):793. 10.3390/cells8080793 PMC6721558 31366062

[134] Krumova K, Cosa G. Chapter 1 overview of reactive oxygen species. In: Nonell S, Flors C, editors. Singlet oxygen: applications in biosciences and nanosciences, volume 1. The Royal Society of Chemistry. Washington DC: Royal Society of Chemistry; 2016. 1–21. doi:10.1039/9781782622208-00001

[135] Plaetzer K, Krammer B, Berlanda J, et al. Photophysics and photochemistry of photodynamic therapy: fundamental aspects. Lasers Med Sci. 2009;24(2):259–268. 18247081 10.1007/s10103-008-0539-1

[136] Valeur B. Molecular fluorescence: principles and applications. Weinheim (Germany): Wiley-VCH; 2001.

[137] Ochsner M. Photophysical and photobiological processes in the photodynamic therapy of tumours. J Photochem Photobiol B. 1997;39(1):1–18. 9210318 10.1016/s1011-1344(96)07428-3

[138] Abrahamse H, Hamblin MR. New photosensitizers for photodynamic therapy. Biochem J. 2016;473(4):347–364. 26862179 10.1042/BJ20150942 PMC4811612

[139] Baier J, Maier M, Engl R, et al. Time-resolved investigations of singlet oxygen luminescence in water, in phosphatidylcholine, and in aqueous suspensions of phosphatidylcholine or HT29 cells. J Phys Chem A. 2005;109(7):3041–3046. 10.1021/jp0455531 16851318

[140] Maisch T, Baier J, Franz B, et al. 2007. The role of singlet oxygen and oxygen concentration in photodynamic inactivation of bacteria. Proc Natl Acad Sci U S A. 104(17):7223–7228. 17431036 10.1073/pnas.0611328104 PMC1851884

[141] Foote CS. Definition of type I and type II photosensitized oxidation. Photochem Photobiol. 1991;54(5):659. 1798741 10.1111/j.1751-1097.1991.tb02071.x

[142] Dąbrowski JM. Chapter nine - reactive oxygen species in photodynamic therapy: mechanisms of their generation and potentiation. In: van Eldik R, Hubbard CD, editors. Advances in inorganic chemistry. Cambridge (Massachusetts): Academic Press; 2017. p. 343–394.

[143] Niedre M, Patterson MS, Wilson BC. Direct near-infrared luminescence detection of singlet oxygen generated by photodynamic therapy in cells in vitro and tissues in vivo. Photochem Photobiol. 2002;75(4):382–391. 12003128 10.1562/0031-8655(2002)075<0382:DNILDO>2.0.CO;2

[144] Boegheim JP, Dubbelman TM, Mullenders LH, et al. Photodynamic effects of haematoporphyrin derivative on DNA repair in murine L929 fibroblasts. Biochem J. 1987;244(3):711–715. 2965572 10.1042/bj2440711 PMC1148054

[145] Huang L, Krayer M, Roubil JGS, et al. Stable synthetic mono-substituted cationic bacteriochlorins mediate selective broad-spectrum photoinactivation of drug-resistant pathogens at nanomolar concentrations. J Photochem Photobiol B Biol. 2014;141:119–127. 10.1016/j.jphotobiol.2014.09.016 PMC4314371 25463659

[146] Weishaupt KR, Gomer CJ, Dougherty TJ. Identification of singlet oxygen as the cytotoxic agent in photo-inactivation of a murine tumor. Cancer Res. 1976;36(7 Part 1):2326–2329. 1277137

[147] Kashef N, Hamblin MR. Can microbial cells develop resistance to oxidative stress in antimicrobial photodynamic inactivation? Drug Resist Updat. 2017;31:31–42. 28867242 10.1016/j.drup.2017.07.003 PMC5673603

[148] Maisch T. Resistance in antimicrobial photodynamic inactivation of bacteria. Photochem Photobiol Sci. 2015;14(8):1518–1526. 26098395 10.1039/c5pp00037h

[149] Alves E, Faustino MA, Neves MG, et al. An insight on bacterial cellular targets of photodynamic inactivation. Future Med Chem. 2014;6(2):141–164. 24467241 10.4155/fmc.13.211

[150] Krammer B, Plaetzer K. ALA and its clinical impact, from bench to bedside. Photochem Photobiol Sci. 2008;7(3):283–289. 18389144 10.1039/b712847a

[151] St Denis TG, Hamblin MR. 22 - Supramolecular drug delivery platforms in photodynamic therapy. In: Hamblin MR, Avci P, editors. Applications of nanoscience in photomedicine. Oxford: Chandos Publishing; 2015. p. 465–485.

[152] Yoo JO, Ha KS. New insights into the mechanisms for photodynamic therapy-induced cancer cell death. Int Rev Cell Mol Biol. 2012;295:139–174. 22449489 10.1016/B978-0-12-394306-4.00010-1

[153] Allison RR, Sibata CH. Oncologic photodynamic therapy photosensitizers: a clinical review. Photodiagnosis Photodyn Ther. 2010;7(2):61–75. 20510301 10.1016/j.pdpdt.2010.02.001

[154] Sobczyński J, Polski A. Chapter 21 - nanocarriers for photosensitizers for use in antimicrobial photodynamic therapy. In: Ficai A, Grumezescu AM, editors. Nanostructures for antimicrobial therapy. Amsterdam (Netherlands): Elsevier; 2017. p. 481–502.

[155] Nanashima A, Nagayasu T. Current status of photodynamic therapy in digestive tract carcinoma in Japan. Int J Mol Sci. 2015;16(2):3434–3440. 25690028 10.3390/ijms16023434 PMC4346905

[156] Lazzeri D, Rovera M, Pascual L, et al. Photodynamic studies and photoinactivation of escherichia coli using meso-substituted cationic porphyrin derivatives with asymmetric charge distribution. Photochem Photobiol. 2004;80(2):286–293. 15362952 10.1562/2004-03-08-RA-105

[157] Rossoni RD, Junqueira JC, Santos EL, et al. Comparison of the efficacy of rose bengal and erythrosin in photodynamic therapy against enterobacteriaceae. Lasers Med Sci. 2010;25(4):581–586. 20232222 10.1007/s10103-010-0765-1

[158] Rajesh S, Koshi E, Philip K, et al. Antimicrobial photodynamic therapy: An overview. Journal of Indian Society of Periodontology, 2011;15 1 323–327. doi:10.4103/0972-124X.92563 22368354 PMC3283927

[159] Dai T, Huang-Y-Y, Hamblin MR. Photodynamic therapy for localized infections–state of the art. Photodiagnosis Photodyn Ther. 2009;6(3–4):170–188. 19932449 10.1016/j.pdpdt.2009.10.008 PMC2811240

[160] Schastak S, Ziganshyna S, Gitter B, et al. Efficient photodynamic therapy against gram-positive and gram-negative bacteria using THPTS, a cationic photosensitizer excited by infrared wavelength. PloS One. 2010;5(7):e11674–e11674. 20652031 10.1371/journal.pone.0011674 PMC2907405

[161] Calzavara-Pinton P, Rossi MT, Sala R, et al. Photodynamic antifungal chemotherapy. Photochem Photobiol. 2012;88(3):512–522. 22313493 10.1111/j.1751-1097.2012.01107.x

[162] Bertoloni G, Zambotto F, Conventi L, et al. Role of specific cellular targets in the hematoporphyrin-sensitized photoinactivation of microbial cells. Photochem Photobiol. 1987;46(5):695–698. 3327061 10.1111/j.1751-1097.1987.tb04834.x

[163] Donnelly RF, McCarron PA, Tunney MM. Antifungal photodynamic therapy. Microbiol Res. 2008;163(1):1–12. 18037279 10.1016/j.micres.2007.08.001

[164] Gad F, Zahra T, Hasan T, et al. Effects of growth phase and extracellular slime on photodynamic inactivation of gram-positive pathogenic bacteria. Antimicrob Agents Chemother. 2004;48(6):2173–2178. 15155218 10.1128/AAC.48.6.2173-2178.2004 PMC415578

[165] Lopes M, Alves CT, Rama Raju B, et al. Application of benzo[a]phenoxazinium chlorides in antimicrobial photodynamic therapy of Candida albicans biofilms. J Photochem Photobiol B Biol. 2014b;141:93–99. 10.1016/j.jphotobiol.2014.09.006 25463655

[166] Bertoloni G, Lauro FM, Cortella G, et al. Photosensitizing activity of hematoporphyrin on Staphylococcus aureus cells. Biochim Biophys Acta. 2000;1475(2):169–174. 10832032 10.1016/s0304-4165(00)00071-4

[167] Lam M, Jou PC, Lattif AA, et al. Photodynamic therapy with Pc 4 induces apoptosis of candida albicans. Photochem Photobiol. 2011a;87(4):904–909. 21521233 10.1111/j.1751-1097.2011.00938.x PMC3139787

[168] Goosen N, Moolenaar GF. Repair of UV damage in bacteria. DNA Repair (Amst). 2008;7(3):353–379. 17951115 10.1016/j.dnarep.2007.09.002

[169] Gracanin M, Hawkins CL, Pattison DI, et al. Singlet-oxygen-mediated amino acid and protein oxidation: formation of tryptophan peroxides and decomposition products. Free Radic Biol Med. 2009;47(1):92–102. 19375501 10.1016/j.freeradbiomed.2009.04.015

[170] Zanin IC, Gonçalves RB, Junior AB, et al. Susceptibility of Streptococcus mutans biofilms to photodynamic therapy: an in vitro study. J Antimicrob Chemother. 2005;56(2):324–330. 15983029 10.1093/jac/dki232

[171] Tegos GP, Demidova TN, Arcila-Lopez D, et al. Cationic fullerenes are effective and selective antimicrobial photosensitizers. Chem Biol. 2005;12(10):1127–1135. 16242655 10.1016/j.chembiol.2005.08.014 PMC3071678

[172] Mahmoudi H, Bahador A, Pourhajibagher M, et al. Antimicrobial photodynamic therapy: an effective alternative approach to control bacterial infections. J Lasers Med Sci. 2018;9(3):154–160. 30809325 10.15171/jlms.2018.29 PMC6378356

[173] Afonso SG, Enríquez de Salamanca R, Batlle AM del C. The photodynamic and non-photodynamic actions of porphyrins. Brazilian Journal of Medical and Biological Research. 1999;32(3):255–266. doi:10.1590/S0100-879X1999000300002 10347781

[174] Wainwright M. Phenothiazinium photosensitisers: v. Photobactericidal activities of chromophore-methylated phenothiazinium salts. Dyes Pigm. 2007;73(1):7–12.

[175] Jesus Tedesco A Low P. level energy photodynamic therapy for skin processes and regeneration. In: Tanaka Y, editor. Photomedicine—Advances in Practice Clinical; Tanaka, Y., Ed.London (UK): IntechOpen; 2017. Volume 5, pp. 75–94. ISBN 978-953-51-3156–4

[176] Pellosi DS, Batistela VR, Souza VR, et al. Evaluation of the photodynamic activity of xanthene dyes on Artemia salina described by chemometric approaches. An Acad Bras Cienc. 2013;85(4):1267–1274. doi:10.1590/0001-3765201395412 24173104

[177] Jendželovská Z, Jendželovský R, Kuchárová B, et al. Hypericin in the light and in the dark: two sides of the same coin. Front Plant Sci. 2016;6(7):560. doi:10.3389/fpls.2016.00560 PMC4859072 27200034

[178] Soukos NS, Wilson M, Burns T, et al. Photodynamic effects of toluidine blue on human oral keratinocytes and fibroblasts and Streptococcus sanguis evaluated in vitro. Lasers Surg Med. 1996;18(3):253–259. 8778520 10.1002/(SICI)1096-9101(1996)18:3<253::AID-LSM6>3.0.CO;2-R

[179] Verma S, Sallum UW, Athar H, et al. Antimicrobial photodynamic efficacy of side-chain functionalized benzo[a]phenothiazinium dyes. Photochem Photobiol. 2009;85(1):111–118. 18657053 10.1111/j.1751-1097.2008.00403.x

[180] Wainwright M, O’Kane C, Rawthore S. Phenothiazinium photosensitisers XI. Improved toluidine blue photoantimicrobials. J Photochem Photobiol B Biol. 2016;160:68–71. 10.1016/j.jphotobiol.2016.03.035 27093001

[181] Encinas MV, Rufs AM, Bertolotti SG, et al. Xanthene dyes/amine as photoinitiators of radical polymerization: a comparative and photochemical study in aqueous medium. Polymer. 2009;50(13):2762–2767.

[182] Sebrão CC, Bezerra AG Jr., de França PH, et al. Comparison of the efficiency of rose bengal and methylene blue as photosensitizers in photodynamic therapy techniques for enterococcus faecalis inactivation. Photomed Laser Surg. 2017;35(1):18–23. 27617768 10.1089/pho.2015.3995

[183] Silva AF, Borges A, Freitas CF, et al. Antimicrobial photodynamic inactivation mediated by rose bengal and erythrosine is effective in the control of food-related bacteria in planktonic and biofilm States. Molecules. 2018;23:9. 10.3390/molecules23092288 PMC6225188 30205468

[184] Paramanantham P, Antony AP, Sruthil Lal SB, et al. Antimicrobial photodynamic inactivation of fungal biofilm using amino functionalized mesoporus silica-rose bengal nanoconjugate against Candida albicans. Sci Afr. 2018;1:e00007

[185] García I, Ballesta S, Gilaberte Y, et al. Antimicrobial photodynamic activity of hypericin against methicillin-susceptible and resistant Staphylococcus aureus biofilms. Future Microbiol. 2015;10(3):347–356. 25812458 10.2217/fmb.14.114

[186] Li B, Li X, Lin H, et al. Curcumin as a promising antibacterial agent: effects on metabolism and biofilm formation in S. mutans. Biomed Res Int. 2018;(2018:4508709. 29682545 10.1155/2018/4508709 PMC5851298

[187] Taha M, Culibrk B, Kalab M, et al. Efficiency of riboflavin and ultraviolet light treatment against high levels of biofilm-derived Staphylococcus epidermidis in buffy coat platelet concentrates. Vox Sang. 2017;112(5):408–416. 28378343 10.1111/vox.12519

[188] Nakajima N, Kawashima N. A basic study on hypericin-PDT in vitro. Photodiagnosis Photodyn Ther. 2012;9(3):196–203. 22959799 10.1016/j.pdpdt.2012.01.008

[189] Lima AM, Pizzol CD, Monteiro FB, et al. Hypericin encapsulated in solid lipid nanoparticles: phototoxicity and photodynamic efficiency. J Photochem Photobiol B. 2013;125:146–154. 23816959 10.1016/j.jphotobiol.2013.05.010

[190] Zhang K, Gao S, Guo J, et al. Hypericin-photodynamic therapy inhibits proliferation and induces apoptosis in human rheumatoid arthritis fibroblast-like synoviocytes cell line MH7A. Iran J Basic Med Sci. 2018b;21(2):130–137. 29456809 10.22038/IJBMS.2018.23871.5991 PMC5811751

[191] Maisch T, Eichner A, Späth A, et al. Fast and effective photodynamic inactivation of multiresistant bacteria by cationic riboflavin derivatives. PLoS One. 2014;9(12):e111792. 25469700 10.1371/journal.pone.0111792 PMC4254278

[192] Kazantzis KT, Koutsonikoli K, Mavroidi B, et al. Curcumin derivatives as photosensitizers in photodynamic therapy: photophysical properties and in vitro studies with prostate cancer cells. Photochem Photobiol Sci Off J Eur Photochem Assoc Eur Soc Photobiol. 2020;19(2):193–206. 10.1039/c9pp00375d 31956888

[193] Spaeth A, Graeler A, Maisch T, et al. CureCuma–cationic curcuminoids with improved properties and enhanced antimicrobial photodynamic activity. Eur J Med Chem. 2018;159:423–440. 29331487 10.1016/j.ejmech.2017.09.072

[194] Cardoso DR, Libardi SH, Skibsted LH. Riboflavin as a photosensitizer. Effects on human health and food quality. Food Funct. 2012;3(5):487–502. doi:10.1039/c2fo10246c 22406738

[195] Dias LD, Blanco KC, Mfouo-Tynga IS, et al. Curcumin as a photosensitizer: from molecular structure to recent advances in antimicrobial photodynamic therapy. J Photochem Photobiol C Photochem Rev. 2020;45:100384.

[196] Mazzone G, Alberto ME, De Simone BC, et al. Can expanded bacteriochlorins act as photosensitizers in photodynamic therapy? Good news from density functional theory computations. Molecules. 2016;21(3):288-288. 10.3390/molecules21030288 PMC6273748 26938516

[197] Saavedra R, Rocha LB, Dąbrowski JM, et al. Modulation of biodistribution, pharmacokinetics, and photosensitivity with the delivery vehicle of a bacteriochlorin photosensitizer for photodynamic therapy. ChemMedChem. 2014;9(2):390–398. 24376035 10.1002/cmdc.201300449

[198] Dovigo LN, Carmello JC, Carvalho MT, et al. Photodynamic inactivation of clinical isolates of Candida using photodithazine®. Biofouling. 2013b;29(9):1057–1067. 24025068 10.1080/08927014.2013.827668

[199] Nyamu SN, Ombaka L, Masika E, et al. Antimicrobial photodynamic activity of phthalocyanine derivatives. Adv Chem. 2018;(2018:2598062.

[200] Braik M, Dridi C, Ben Ali M, et al. Development of a capacitive chemical sensor based on Co(II)-phthalocyanine acrylate-polymer/HfO2/SiO2/Si for detection of perchlorate. J Sens Sens Syst. 2015;4(1):17–23.

[201] Ertem B, Bilgin A, Gök Y, et al. The synthesis and characterization of novel metal-free and metallophthalocyanines bearing eight 16-membered macrocycles. Dyes Pigm. 2008;77(3):537–544.

[202] Ahmad A, Rahman HA, Khan MU. Phthalocyanines derivatives as control approach for antimicrobial photodynamic therapy. J. Am J Clin Microbiol Antimicrob. 2019;2(3):1–8.

[203] Perni S, Prokopovich P, Pratten J, et al. Nanoparticles: their potential use in antibacterial photodynamic therapy. Photochem Photobiol Sci. 2011;10(5):712–720. 21380441 10.1039/c0pp00360c

[204] Sadasivam M, Avci P, Gupta GK, et al. Self-assembled liposomal nanoparticles in photodynamic therapy. Eur J Nanomed. 2013;5(3). DOI:10.1515/ejnm-2013-0010 PMC3857307 24348377

[205] Yamakoshi Y, Umezawa N, Ryu A, et al. Active oxygen species generated from photoexcited fullerene (C60) as potential medicines: O2-* versus 1O2. J Am Chem Soc. 2003;125(42):12803–12809. 14558828 10.1021/ja0355574

[206] Mehraban N, Freeman HS. Developments in PDT sensitizers for increased selectivity and singlet oxygen production. Materials (Basel). 2015;8(7):4421–4456. 28793448 10.3390/ma8074421 PMC5455656

[207] Mroz P, Tegos GP, Gali H, et al. Photodynamic therapy with fullerenes. Photochem Photobiol Sci. 2007;6(11):1139–1149. 17973044 10.1039/b711141j PMC2933422

[208] Kim YW, Subramanian S, Gerasopoulos K, et al. Effect of electrical energy on the efficacy of biofilm treatment using the bioelectric effect. NPJ Biofilms Microbiomes. 2015;1(1):15016. 28721233 10.1038/npjbiofilms.2015.16 PMC5515217

[209] Wilson BC, Patterson MS. The physics, biophysics and technology of photodynamic therapy. Phys Med Biol. 2008;53(9):R61–109. 18401068 10.1088/0031-9155/53/9/R01

[210] Drosdoff D, Widom A. Snell’s law from an elementary particle viewpoint. Am J Phys. 2005;73(10):973–975.

[211] Kobe D, Spaniol S, Beyer W, et al. Computer modeling of light distributions for diverse photodynamic therapy applicators. Bellingham (United States): SPIE; 1994.

[212] Niemz M. Laser-Tissue Interactions: fundamentals and Applications. Berlin (Germany): Springer-Verlag Berlin Heidelberg; 2002.

[213] Prasad PN. Introduction to biophotonics. Hoboken: Wiley; 2003.

[214] MH N. Laser-tissue interactions. Fundamentals and applications. Berlin Heidelberg New York: Springer; 2004.

[215] Castano AP, Demidova TN, Hamblin MR. Mechanisms in photodynamic therapy: part one-photosensitizers, photochemistry and cellular localization. Photodiagnosis Photodyn Ther. 2004;1(4):279–293. 25048432 10.1016/S1572-1000(05)00007-4 PMC4108220

[216] Rajesh S, Koshi E, Philip K, et al. Antimicrobial photodynamic therapy: an overview. J Indian Soc Periodontol. 2011b;15(4):323–327. 22368354 10.4103/0972-124X.92563 PMC3283927

[217] Salva KA. Photodynamic therapy: unapproved uses, dosages, or indications. Clin Dermatol. 2002;20(5):571–581. 12435528 10.1016/s0738-081x(02)00266-3

[218] Rosa LP,DSF. Antimicrobial photodynamic therapy: a new therapeutic option to combat infections. J Med Microbiol Diagn. 2014;158(3). DOI:10.4172/2161-0703.1000158

[219] Nagata JY, Hioka N, Kimura E, et al. Antibacterial photodynamic therapy for dental caries: evaluation of the photosensitizers used and light source properties. Photodiagnosis Photodyn Ther. 2012;9(2):122–131. 22594982 10.1016/j.pdpdt.2011.11.006

[220] Schubert E. Light-emitting diodes. Cambridge: Cambridge University; 2006.

[221] Szeimies RM, Hein R, Bäumler W, et al. A possible new incoherent lamp for photodynamic treatment of superficial skin lesions. Acta Derm Venereol. 1994;74(2):117–119. 7911616 10.2340/0001555574117119

[222] Spranley TJ, Winkler M, Dagate J, et al. Curing light burns. Gen Dent. 2012;60(4):e210–214. 22782053

[223] Wood S, Metcalf D, Devine D, et al. Erythrosine is a potential photosensitizer for the photodynamic therapy of oral plaque biofilms. J Antimicrob Chemother. 2006;57(4):680–684. 16464894 10.1093/jac/dkl021

[224] Sterenborg HJCM, Gemert MJCV. Photodynamic therapy with pulsed light sources: a theoretical analysis. Phys Med Biol. 1996;41(5):835–849. 8735252 10.1088/0031-9155/41/5/002

[225] Schoenbach KH, Richard Heller EN, Thomas P, et al. Introduction. In: Heller HAAR, editor. Bioeletrics. Japan: Springer; 2017. p. 1–40.

[226] Kotnik T, Rems L, Tarek M, et al. Membrane electroporation and electropermeabilization: mechanisms and models. Annu Rev Biophys. 2019;48:63–91. 30786231 10.1146/annurev-biophys-052118-115451

[227] Stewart MP, Langer R, Jensen KF. Intracellular delivery by membrane disruption: mechanisms, strategies, and concepts. Chem Rev. 2018;118(16):7409–7531. 30052023 10.1021/acs.chemrev.7b00678 PMC6763210

[228] Stankevic V, Šimonis P, Zurauskiene N, et al. Compact square-wave pulse electroporator with controlled electroporation efficiency and cell viability. Symmetry. 2020;12:412.

[229] Gabriel B, TEISSIÉ J. Generation of reactive-oxygen species induced by electropermeabilization of Chinese hamster ovary cells and their consequence on cell viability. Eur J Biochem. 1994;223(1):25–33. 8033899 10.1111/j.1432-1033.1994.tb18962.x

[230] Maccarrone M, Bladergroen MR, Rosato N, et al. Role of lipid peroxidation in electroporation-induced cell permeability. Biochem Biophys Res Commun. 1995;209(2):417–425. 7733908 10.1006/bbrc.1995.1519

[231] Galutzov B, Ganeva V. Lucigenin-derived chemiluminescence in Saccharomyces cerevisiae. Bioelectrochem Bioenerg. 1997;44(1):77–82.

[232] Bonnafous P, Vernhes M-C, Teissié J, et al. The generation of reactive-oxygen species associated with long-lasting pulse-induced electropermeabilisation of mammalian cells is based on a non-destructive alteration of the plasma membrane. Biochim Biophys Acta - Biomembr. 1999;1461(1):123–134. 10.1016/s0005-2736(99)00154-6 10556494

[233] Michel O, Pakhomov AG, Casciola M, et al. Electropermeabilization does not correlate with plasma membrane lipid oxidation. Bioelectrochemistry. 2020;132:107433. 31891877 10.1016/j.bioelechem.2019.107433

[234] Pakhomova ON, Khorokhorina VA, Bowman AM, et al. Oxidative effects of nanosecond pulsed electric field exposure in cells and cell-free media. Arch Biochem Biophys. 2012;527(1):55–64. 22910297 10.1016/j.abb.2012.08.004 PMC3459148

[235] Panday A, Sahoo MK, Osorio D, et al. NADPH oxidases: an overview from structure to innate immunity-associated pathologies. Cell Mol Immunol. 2015;12(1):5–23. 25263488 10.1038/cmi.2014.89 PMC4654378

[236] Jiang F, Zhang Y, Dusting GJ. NADPH oxidase-mediated redox signaling: roles in cellular stress response, stress tolerance, and tissue repair. Pharmacol Rev. 2011;63(1):218–242. 21228261 10.1124/pr.110.002980

[237] Kašėta V, Kaušylė A, Kavaliauskaitė J, et al. Detection of intracellular biomarkers in viable cells using millisecond pulsed electric fields. Exp Cell Res. 2020;389(1):111877. 31991124 10.1016/j.yexcr.2020.111877

[238] Khlupova M, Kuznetsov B, Gonchar M, et al. Amperometric monitoring of redox activity in intact, permeabilised and lyophilised cells of the yeast Hansenula polymorpha. Electrochem commun. 2007;9(7):1480–1485.

[239] Simonis P, Garjonyte R, Stirke A. Mediated amperometry as a prospective method for the investigation of electroporation. Sci Rep. 2020;10(1):19094. 33154473 10.1038/s41598-020-76086-2 PMC7644768

[240] Beebe SJ, Chen Y-J, Sain NM, et al. Transient features in nanosecond pulsed electric fields differentially modulate mitochondria and viability. PLOS ONE. 2012;7(12):e51349. 23284682 10.1371/journal.pone.0051349 PMC3528752

[241] Pakhomov AG, Gianulis E, Vernier PT, et al. Multiple nanosecond electric pulses increase the number but not the size of long-lived nanopores in the cell membrane. Biochim Biophys Acta - Biomembr. 2015;1848(4):958–966. 10.1016/j.bbamem.2014.12.026 PMC4331219 25585279

[242] Nuccitelli R, Lui K, Kreis M, et al. Nanosecond pulsed electric field stimulation of reactive oxygen species in human pancreatic cancer cells is Ca2+-dependent. Biochem Biophys Res Commun. 2013;435(4):580–585. 23680664 10.1016/j.bbrc.2013.05.014 PMC3730523

[243] Simonis P, Kersulis S, Stankevich V, et al. Caspase dependent apoptosis induced in yeast cells by nanosecond pulsed electric fields. Bioelectrochemistry. 2017;115:19–25. 28236755 10.1016/j.bioelechem.2017.01.005

[244] Stirke A, Zimkus A, Balevicius S, et al. Permeabilization of yeast Saccharomyces cerevisiae cell walls using nanosecond high power electrical pulses. Appl Phys Lett. 2014;105(25):253701.

[245] Sato T, Machida T, Takahashi S, et al. Fas-mediated apoptosome formation is dependent on reactive oxygen species derived from mitochondrial permeability transition in jurkat cells. J Immunol. 2004;173(1):285–296. 15210786 10.4049/jimmunol.173.1.285

[246] Hamada Y, Furumoto Y, Izutani A, et al. Nanosecond pulsed electric fields induce the integrated stress response via reactive oxygen species-mediated heme-regulated inhibitor (HRI) activation. PLOS ONE. 2020;15(3):e0229948. 32155190 10.1371/journal.pone.0229948 PMC7064201

[247] Saulis G, Rodaitė‐Riševičienė R, Dainauskaitė VS, et al. Electrochemical processes during high-voltage electric pulses and their importance in food processing technology. In: R.r. V., editor. Advances in food biotechnology. Hoboken (New Jersey): John Wiley & Sons; 2015. p. 575–592.

[248] Rodaitė-Riševičienė R, Saulė R, Snitka V, et al. Release of iron ions from the stainless steel anode occurring during high-voltage pulses and its consequences for cell electroporation technology. IEEE Trans Plasma Sci. 2014;42(1):249–254.

[249] Ruzgys P, Novickij V, Novickij J, et al. Influence of the electrode material on ROS generation and electroporation efficiency in low and high frequency nanosecond pulse range. Bioelectrochemistry. 2019;127:87–93. 30769178 10.1016/j.bioelechem.2019.02.002

[250] Teissie J. Involvement of reactive oxygen species in membrane electropermeabilization. In: Miklavcic D, editor. Handbook of Electroporation. Cham: Springer International Publishing; 2017. p. 1–15.

[251] Muñoz-Mahamud E, González-Cuevas A, Sierra JM, et al. Pulsed electric fields reduce bacterial attachment to stainless steel plates. Acta Orthop Belg. 2018;84(1):11–16. 30457494

[252] Perez-Roa RE, Tompkins DT, Paulose M, et al. Effects of localised, low-voltage pulsed electric fields on the development and inhibition of Pseudomonas aeruginosa biofilms. Biofouling. 2006;22(5–6):383–390. 17178571 10.1080/08927010601053541

[253] Khan SI, Blumrosen G, Vecchio D, et al. Eradication of multidrug-resistant pseudomonas biofilm with pulsed electric fields. Biotechnol Bioeng. 2016;113(3):643–650. 26332437 10.1002/bit.25818 PMC4729586

[254] Novickij V, Zinkevičienė A, Perminaitė E, et al. Non-invasive nanosecond electroporation for biocontrol of surface infections: an in vivo study. Sci Rep. 2018;8(1):14516. 30266920 10.1038/s41598-018-32783-7 PMC6162327

[255] Blenkinsopp SA, Khoury AE, Costerton JW. Electrical enhancement of biocide efficacy against Pseudomonas aeruginosa biofilms. Appl Environ Microbiol. 1992;58(11):3770–3773. 1482196 10.1128/aem.58.11.3770-3773.1992 PMC183173

[256] Khoury AE, Lam K, Ellis B, et al. Prevention and control of bacterial infections associated with medical devices. Asaio J. 1992;38(3):M174–178. 1457842 10.1097/00002480-199207000-00013

[257] Stewart PS, Wattanakaroon W, Goodrum L, et al. Electrolytic generation of oxygen partially explains electrical enhancement of tobramycin efficacy against Pseudomonas aeruginosa biofilm. Antimicrob Agents Chemother. 1999;43(2):292–296. 9925521 10.1128/aac.43.2.292 PMC89066

[258] Costerton JW, Ellis B, Lam K, et al. Mechanism of electrical enhancement of efficacy of antibiotics in killing biofilm bacteria. Antimicrob Agents Chemother. 1994;38(12):2803–2809. 7695266 10.1128/aac.38.12.2803 PMC188289

[259] Stirke A, Celiesiute-Germaniene R, Zimkus A, et al. The link between yeast cell wall porosity and plasma membrane permeability after PEF treatment. Sci Rep. 2019;9(1):14731. 31611587 10.1038/s41598-019-51184-y PMC6791849

[260] Alves E, Costa L, Carvalho CM, et al. Charge effect on the photoinactivation of Gram-negative and Gram-positive bacteria by cationic meso-substituted porphyrins. BMC Microbiol. 2009;9:70. 19368706 10.1186/1471-2180-9-70 PMC2672088

[261] Engel E, Schraml RD, Maisch T, et al. Light-induced decomposition of indocyanine green. Invest Ophthalmol Vis Sci. 2008;49(5):1777–1783. 18436812 10.1167/iovs.07-0911

[262] Soukos NS, Goodson JM. Photodynamic therapy in the control of oral biofilms. Periodontol 2000. 2011;55(1):143–166. 21134233 10.1111/j.1600-0757.2010.00346.x

[263] Michael Ngadi XJ, Smith J, Raghavan GSV. Inactivation of escherichia coli O157: H7 in poultry chiller water using combined ultraviolet light, pulsed electric field and ozone treatments. Int J Poult Sci. 2004;3(11):733–737.

[264] Palgan I, Caminiti IM, Muñoz A, et al. Combined effect of selected non-thermal technologies on Escherichia coli and Pichia fermentans inactivation in an apple and cranberry juice blend and on product shelf life. Int J Food Microbiol. 2011;151(1):1–6. 21893360 10.1016/j.ijfoodmicro.2011.07.019

[265] Cebrián G, Mañas P, Condón S. Comparative resistance of bacterial foodborne pathogens to non-thermal technologies for food preservation. Front Microbiol. 2016;7:734. 27242749 10.3389/fmicb.2016.00734 PMC4873515

[266] Kulbacka J. Nanosecond pulsed electric fields (nsPEFs) impact and enhanced Photofrin II® delivery in photodynamic reaction in cancer and normal cells. Photodiagnosis Photodyn Ther. 2015;12(4):621–629. 26563460 10.1016/j.pdpdt.2015.11.002

[267] Zhang H, Liu K, Xue Z, et al. High-voltage pulsed electric field plus photodynamic therapy kills breast cancer cells by triggering apoptosis. Am J Transl Res. 2018a;10(2):334–351. 29511429 PMC5835800

